# Endothelial HSPA12B regulates myocardial monocyte infiltration and inflammatory activity after myocardial infarction

**DOI:** 10.3389/fimmu.2025.1587898

**Published:** 2025-05-15

**Authors:** Yana Wang, Min Fan, Linjian Chen, Patrick Spencer Gill, Xiaohui Wang, Tuanzhu Ha, David L. Williams, Chuanfu Li, Kun Yang

**Affiliations:** ^1^ Department of Surgery, James H. Quillen College of Medicine, East Tennessee State University, Johnson City, TN, United States; ^2^ Center of Excellence in Inflammation, Infectious Disease and Immunity, East Tennessee State University, Johnson City, TN, United States

**Keywords:** myocardial infarction, endothelial cells, HSPA12B, exosome, cardiac resident macrophages, cardiac infiltrated monocytes

## Abstract

**Introduction:**

Cardiac macrophages are essential mediators of inflammation and tissue remodeling following myocardial infarction (MI). Endothelial cell-specific heat shock protein A12B (eHSPA12B) has emerged as a key vascular regulator, but its role in modulating immune cell responses after MI remains unknown. This study investigates whether eHSPA12B regulates monocyte infiltration following MI injury.

**Methods:**

We used endothelial cell-specific Hspa12b knockout (e*Hspa12b* ^-/-^) and wild-type (WT) mice to assess cardiac function and monocyte infiltration following MI. Cardiac resident macrophages and infiltrating monocytes were examined by flow cytometry 3 days post-MI. Plasma levels of pro-inflammatory cytokines were evaluated by ELISA following MI. To investigate the mechanism by which Hspa12b regulates immune response of macrophages, endothelial cells were transduced with adenovirus expressing HSPA12B followed by hypoxia challenge. In a separate experiment, endothelial cell-derived exosomes were prepared. Macrophages, Raw 264.7 or bone marrow derived macrophages (BMDMs) were incubated with endothelial cell conditioned medium or endothelial cell-derived exosomes. Macrophage phenotypes were examined by immunofluorescence staining, ELISA and qPCR. Protein degradation of toll-like receptor 4 (TLR4) and myeloid differentiation primary response 88 (MyD88) in macrophages was assessed by immunoprecipitation and Western blotting.

**Results:**

e*Hspa12b* ^-/-^ mice exhibited significantly worsened cardiac function and increased infiltration of monocytes compared to WT controls at 3 days post-MI. Conditioned medium from HSPA12B-overexpressing endothelial cells promoted a pro-regenerative macrophage phenotype, characterized by reduced pro-inflammatory and increased anti-inflammatory cytokine production. HSPA12B was secreted via exosomes from endothelial cells, and these exosomes were sufficient to induce macrophage polarization. Mechanistically, uptake of HSPA12B-containing exosomes promotes the degradation of TLR4 and MyD88 in macrophages.

**Discussion:**

Endothelial HSPA12B plays a novel immunomodulatory role in controlling monocyte infiltration and immune activation following MI.

## Introduction

Despite significant improvements in therapeutic interventions, myocardial infarction (MI) continues to be the major cause of heart failure and related mortality in the United States (1–3). Macrophages act as important regulators in response to cardiac injury, including MI (4). In the initial phase of MI, the activation of macrophages contributes to the post-infarction inflammatory response. Typically, infiltrating macrophages, identified by markers such as Ly6C^hi^/CCR2^+^, are crucial components of the innate immune and inflammatory responses following myocardial ischemic injury (5–7). In contrast, cardiac resident macrophages, which are characterized by markers such as Ly6C^-^CD64^+^Tim4^+^, are important for anti-inflammatory responses and the repair process after myocardial ischemic injury (5–7). Previous studies have shown that resident cardiac macrophages undergo programmed cell death and are depleted in response to MI (8, 9). A decrease in resident cardiac monocytes is accompanied by increased infiltration of extracardiac monocytes (5, 9, 10). However, the underlying mechanisms remain unclear.

Accumulating evidence suggests that endothelial cell–immune cell interactions play important roles in regulating tissue repair following cardiac injuries such as MI (11–13). On the one hand, macrophage-derived exosomes and cytokines modulate the proliferation and migration of endothelial cells, thereby orchestrating angiogenesis post-MI (11, 12, 14, 15).

On the other hand, endothelial cells can also regulate macrophage activity within the cardiovascular system (13, 16, 17). A recent study suggested that endothelial cell sphingosine 1-phosphate receptor 1 (S1pr1) attenuates cardiac dysfunction by promoting the accumulation of reparative macrophages after MI (16). Moreover, MI- and ischemia/reperfusion-induced angiopoietin-2 upregulation in endothelial cells is responsible for proinflammatory macrophage polarization in the heart (17). Taken together, these findings suggest that endothelial cells exert a critical role in modulating cardiac macrophage phenotypes, which significantly contributes to cardiac remodeling post-MI.

As a newly discovered member of the heat shock protein 70 (HSP70) family, HSPA12B is predominantly expressed in endothelial cells (18, 19). We and others have demonstrated that HSPA12B is required for angiogenesis both *in vivo* and *in vitro* (19–21). Deficiency of endothelial *Hspa12b* impairs cardiac angiogenesis and cardiac function 4 weeks after MI (20). In contrast, overexpression of HSPA12B mitigates cardiac dysfunction induced by MI via an eNOS-dependent mechanism (22). However, whether endothelial HSPA12B can regulate macrophage activation following MI is unknown. Given the significant influence of endothelial cells on macrophage function, we investigated the impact of endothelial HSPA12B on cardiac macrophage polarization post-MI. We observed that specific deficiency of endothelial cell *Hspa12b* (*Hspa12b* ^-/-^) leads to the accumulation of infiltrated monocytes in the myocardium, thereby exacerbating cardiac dysfunction following MI. *In vitro* studies revealed that endothelial cell HSPA12B can be released via exosome secretion and transmitted to macrophages through exosome uptake, thereby promoting macrophage polarization to the M2 phenotype following hypoxic stimulation. Importantly, exosomes containing HSPA12B facilitate the ubiquitination and degradation of toll-like receptor 4 (TLR4) and myeloid differentiation primary response 88 (MyD88) in macrophages. Correspondingly, endothelial cell HSPA12B ameliorated hypoxia-induced pro-inflammatory cytokine expression and increased anti-inflammatory cytokine production in macrophages. Collectively, we elucidated a novel role for endothelial HSPA12B in regulating cardiac macrophage polarization post-MI *in vivo* and under hypoxia *in vitro*. Augmenting endothelial cell HSPA12B levels could represent a novel strategy for cardiac injury repair following MI.

## Methods

### Animals

Endothelial specific HSPA12B knockout mice (e*Hspa12b* ^-/-^) were generated by cross-breeding the conditionally targeted HSPA12B mice with C57BL/6.Cg-Tg (Tek-cre) strain which carries Cre recombinase under the control of the Tek promoter. e*Hspa12b* ^-/-^ mice and age matched wild type (WT) C57BL/6 mice were used for experiments. The validation of specific knockout of HSPA12B in cardiac endothelial cells were reported in our previous studies (23). Both WT and e*Hspa12b* ^-/-^ mice were raised at the Division of Laboratory Animal Resources at East Tennessee State University (ETSU). All mouse experimental procedures were performed in accordance with the Guide for the Care and Use of Laboratory Animals published by the National Institutes of Health (NIH Publication, 8th Edition, 2011) and approved by the ETSU Committee on Animal Care.

### Myocardial infarction

Eight- to ten-week-old WT and *Hspa12b* ^-/-^ mice were subjected to myocardial infarction (MI) via ligation of the left anterior descending (LAD) coronary artery as described previously (20, 24). In brief, the mice were ventilated after being anesthetized via 5% isoflurane inhalation, and anesthesia was maintained via 2% isoflurane in 100% oxygen. Following a left thoracotomy, the heart was exposed, and the LAD coronary artery was permanently ligated with an 8–0 silk suture. The chest wall incision was then closed, and the mice were allowed to recover in prewarmed cages.

### 2,3,5-Triphenyltetrazolium chloride staining

Infarct size was evaluated by TTC staining as previously described (24). In brief, 6 hours after MI surgery, hearts were collected and cut into 5 slices followed by staining with 0.8% TTC solution (Sigma–Aldrich) at 37 ˚C for 15 minutes.

### Echocardiography

To measure cardiac function, echocardiography was performed on anesthetized mice at baseline and 3 days after MI. The percent fractional shortening (FS%) and percent ejection fraction (EF%) were calculated as described in our previous study (20, 24).

### Immunofluorescence staining

Murine hearts harvested from each group were fixed in 4% paraformaldehyde, embedded in paraffin and cut into 5 mm sections. After deparaffinization, the sections were stained with an anti-F4/80 antibody (1:100 dilution; Abcam, ab6640). *In vitro*, raw 264.7 cells or bone marrow-derived macrophages (BMDMs) were fixed, permeabilized and blocked, followed by staining with anti-CD68, anti-iNOS, anti-Arg-1, or anti-HSPA12B (1:100 dilution; a gift from Zhihua Han, ETSU, Johnson City, Tennessee, USA). DAPI (blue, Vector Laboratories) was used to counterstain the nuclei. The stained sections and cells were measured via a confocal microscope (Leica). For the imaging of cardiac tissue staining, infarct border zone of MI hearts was elected and imaged. To ensure consistency and relevance to injury-associated changes, at least three representative fields were captured from the border zone of each heart. To ensure the unbiased analysis, all image acquisition, zone selection, and quantification were conducted by investigators blinded to the treatment groups to eliminate observer bias.

### Flow cytometry

To examine the phenotypes of macrophages in the myocardium, heart tissues from both WT and *Hspa12b* ^-/-^ mice were digested with 1 mg/mL collagenase I and 1 mg/mL collagenase IV (Sigma–Aldrich) at 37°C as described in our previous study (24). Red blood cell lysis was performed with RBC Lysis Buffer (Santa Cruz, sc-296258). Dead cells were removed by MojoSort Mouse Dead Cell Removal kit (BioLegend, 480157) immediately prior to antibody staining. The cell suspensions were stained with the following antibodies: anti-CD45-PerCP/Cyanine 5.5 (BioLegend, 103132), anti-Ly6G-FITC (B BioLegend, 127606), anti-CCR2-BV421 (BioLegend, 150605), anti-Ly-6C-BV510 (BioLegend, 128033), anti-CD64-APC (BioLegend, 139306), anti-MHC II-APC/Cyanine 7 (BioLegend, 107628), and anti-Tim 4-PE (BioLegend, 130006). Flow cytometry was performed with a BD FACSfortessa flow cytometer (Becton Dickinson), and the results were analyzed with FlowJo software.

### Cell culture

The Raw 264.7 cell line was purchased from ATCC and cultured in Dulbecco’s modified Eagle’s medium (DMEM, Sigma–Aldrich) supplemented with 10% fetal bovine serum (FBS). Bone marrow cells were isolated from femurs and tibias and cultured in DMEM supplemented with penicillin (100 U/mL) and streptomycin (100 ng/mL) as we described previously (25). After the cells were suspended, 10% L929-conditioned medium was used to differentiate the bone marrow cells into BMDMs. The human umbilical vein endothelial cell (HUVEC) cell line was purchased from ATCC and cultured in Vascular Cell Basal Medium (ATCC) supplemented with growth factors (Endothelial Cell Growth Factor Kit-VEGF, ATCC) and 5% FBS. When HUVECs reached 70% confluence, they were transfected with adenovirus expressing HSPA12B labeled with GFP (Ad-HSPA12B) or scrambled GFP (Ad-GFP). Twenty-four hours after transfection, the cells were subjected to hypoxic challenge in a hypoxia chamber (0.1% O_2_, Pro-Ox Model C21, BioSpherix) for 6 hours. The cells incubated under normoxia conditions served as controls. The supernatants were collected as endothelial cell-conditioned medium (ECCM). Raw 264.7 cells or BMDMs were subsequently incubated with ECCM under normoxia or hypoxic conditions for 6 hours. The experimental groups were as follows (1): normoxia-control ECCM + hypoxia-macrophages (2), hypoxia-control ECCM + hypoxia-macrophages (3), hypoxia-Ad-GFP ECCM + hypoxia-macrophages, and (4) hypoxia-Ad-HSPA12B ECCM + hypoxia-macrophages. For some experiments, the cells were treated with the proteasome inhibitor MG-132 (12 μM, Sigma) before they were subjected to hypoxia.

### Enzyme-linked immunosorbent assay

The production of inflammatory cytokines (TNF-α, IL-4, IL-6 and IL-10) in macrophages was examined via commercially available ELISA kits (PeproTech) according to the instructions provided by the manufacturer.

### Isolation of exosomes

Exosomes were isolated from ECCM via a polyethylene glycol (PEG)-based method as previously described (25). Briefly, ECs were cultured with 10% exosome-depleted FBS (Thermo Fisher Scientific). Exosome-containing ECCMs were collected and mixed with 34% PEG 6000 (Sigma) solution overnight, followed by centrifugation. The supernatant was discarded, and the remaining pellet contained the exosomes. The expression of the exosome marker CD63 was confirmed by Western blotting. The quality of exosomes was confirmed by dynamic light scattering using a particle and molecule size analyzer (ZetasizerNano ZS, Malvern Instruments) according to the manufacturer’s instruction (25). The size of exosomes is 125.02 ± 24.6 nm in diameter (25). 4.24 µg/ml exosomes were used for *in vitro* treatment.

### Western blotting and immunoprecipitation

Western blotting was performed as described in previous studies (20, 24, 25). Cellular and exosomal proteins were extracted, and the protein concentration was determined with a BCA Protein Assay Kit (Thermo Fisher Scientific). Western blotting was performed with standard techniques, and the following primary antibodies were used in this study: anti-HSPA12B antibody, 1:1000 dilution; anti-GAPDH antibody, 1:1000 dilution, 2118s, Cell Signaling Technology; anti-CD63 antibody, 1:200 dilution, sc-15363, Santa Cruz; anti-TLR4 antibody, 1:1000 dilution, 14358s, Cell Signaling Technology; anti-MyD88 antibody, 1:1000 dilution, 4283s, Cell Signaling Technology; anti-IKKβ antibody, 1:1000 dilution, 8943s, Cell Signaling Technology; anti-p-NF-κB antibody, 1:1000 dilution, 3033s, Cell Signaling Technology; and anti-NF-κB antibody, 1:1000 dilution, 6956s, Cell Signaling Technology. Ubiquitination of TLR4 and MyD88 was measured by immunoprecipitation as we previously described (20, 25). In brief, 200 µg of protein lysate was incubated with anti-TLR4 or anti-MyD88 antibodies, after which protein A/G-agarose beads (Santa Cruz Biotechnology) were added. The precipitates were washed and boiled in SDS sample buffer. The supernatant was subjected to immunoblotting with an anti-Ub antibody (1:200 dilution, sc-9133, Santa Cruz). The signals were quantified via a G:BOX gel imaging system (Syngene).

### Quantitative real-time PCR

The cellular RNA was extracted via RNAzol RT (Molecular Research Center) in accordance with the manufacturer’s protocol as previously described (20). mRNA was converted to cDNA via a cDNA Reverse Kit (Applied Biosystems). qRT–PCR was performed on a 4800 RT–PCR instrument (Bio-Rad). The mRNA levels of *iNOS*, *Arg-1*, *Tlr4* and *Myd88* were examined via SYBR Green mix (Sigma–Aldrich) and normalized to *Actb*.

### Statistics

All the data in the figures are expressed as the means ± standard deviations. Before statistical analysis, the normality or approximately normality of the data was assessed. Comparisons of data between groups were determined by either t tests or one- or two-way analysis of variance (ANOVA) followed by Tukey’s procedure for multiple range tests, with P < 0.05 considered significant.

## Results

### Deficiency of endothelial *Hspa12b* increases macrophage accumulation in the myocardium and worsens cardiac dysfunction following myocardial infarction

**Figure 1:**
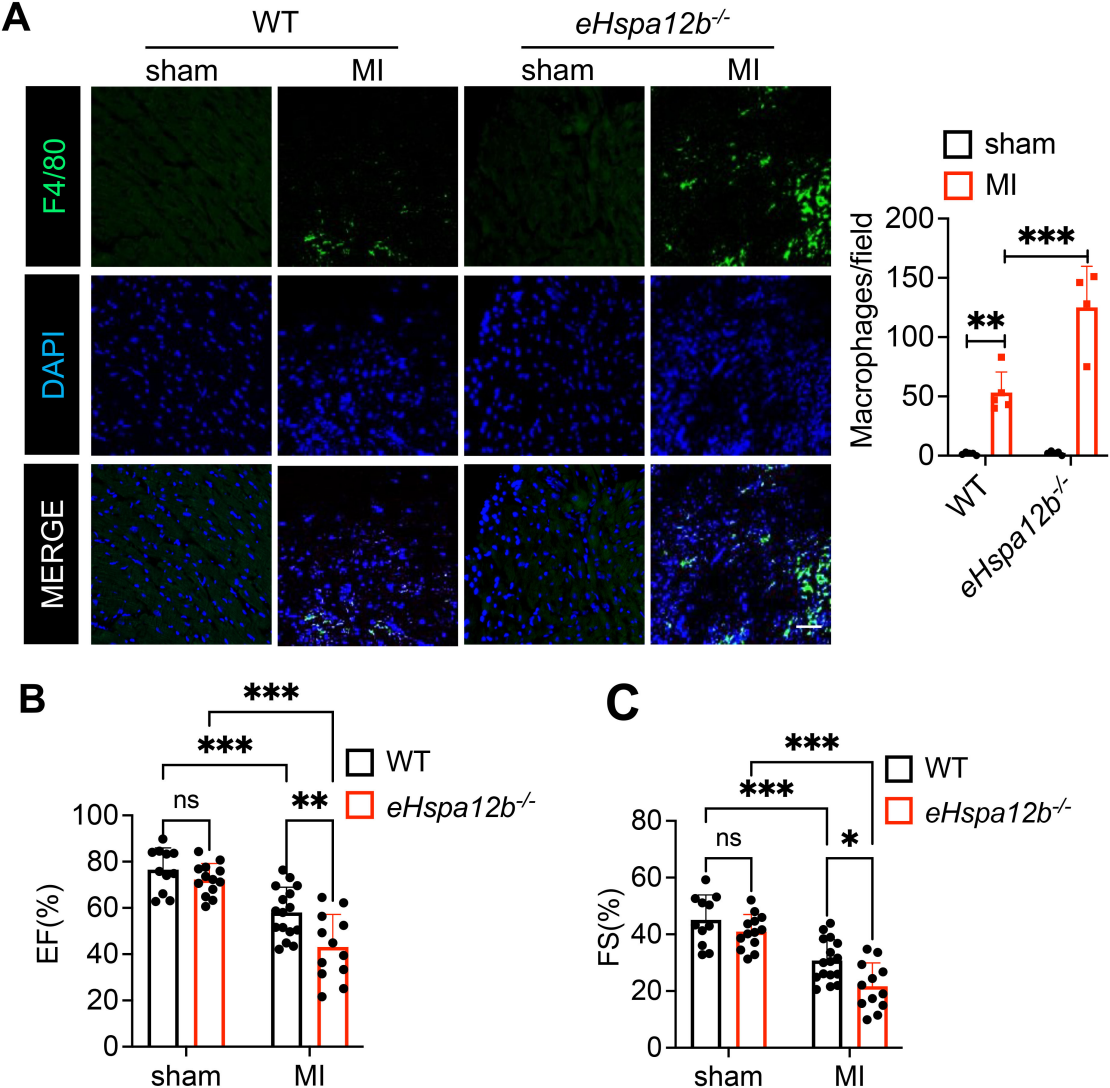
Endothelial *Hspa12b* deficiency increases cardiac macrophage accumulation and exacerbates cardiac dysfunction 3 days after MI. WT and endothelial cell-specific *Hspa12b* knockout (*Hspa12b^-/-^*) mice were subjected to MI or sham surgery. **(A)** Cardiac macrophage accumulation was measured by immunofluorescent staining of heart tissues using anti-F4/80 antibody 3 days post-MI (N=4-5) (Scale bar: 50µm). **(B, C)** Cardiac function was measured by ejection fraction (EF%) and fractional shortening (FS%) at baseline and 3 days after MI (N=11-16). Comparisons of data between groups were made using two-way ANOVA followed by Tukey’s procedure. *P < 0.05, **P < 0.01, ***P < 0.001 compared with indicated groups. ns, no significance.

We previously reported that transgenic mice with endothelial cell-specific overexpression of HSPA12B protect against endotoxin-induced cardiomyopathy via activation of PI3K signaling (26). Recent evidence suggests that the accumulation and activation of macrophages lead to cardiac inflammation, cardiac fibrosis and subsequent tissue damage (27–29). In the present study, we examined whether the protective effect of endothelial cell HSPA12B against myocardial ischemic injury involves the regulation of myocardial macrophage numbers and phenotypes. We induced MI in wild-type (WT) and endothelial *Hspa12b* ^-/-^ mice. Heart tissue was harvested on day 3 post-MI, and immunofluorescence staining of the tissue sections was performed via an anti-F4/80 antibody to detect macrophages in the myocardium. We found that MI injury induced macrophage accumulation in the myocardium (**Figure 1A**) and decreased the ejection fraction (EF%, **Figure 1B**) and fractional shortening (FS%, **Figure 1C**) values, when compared to sham mice. Notably, the number of macrophages in the myocardium of *Hspa12b* ^-/-^ MI mice was significantly greater than that in the myocardium of WT MI mice (**Figure 1A**). In addition, the values of EF% and FS% in *Hspa12b* ^-/-^ MI mice were lower than those in WT MI mice, indicating that *Hspa12b* ^-/-^ mice exhibited greater cardiac dysfunction than WT mice after MI (**Figures 1B, C**). To eliminate the possibility that the observed differences in cardiac function and monocyte infiltration were due to injury severity, we performed TTC staining of injured hearts from both WT and *Hspa12b* ^-/-^ mice 6 hours post-MI induction. As shown in **Supplementary Figure S1**, the infarct sizes between WT and *Hspa12b* ^-/-^ MI hearts were comparable. Therefore, our data suggest that endothelial cell HSPA12B regulates macrophage infiltration into the myocardium, which may subsequently contribute to cardiac dysfunction following MI injury.

### Depletion of endothelial *Hspa12b* promotes monocytes infiltration in the myocardium after MI

**Figure 2:**
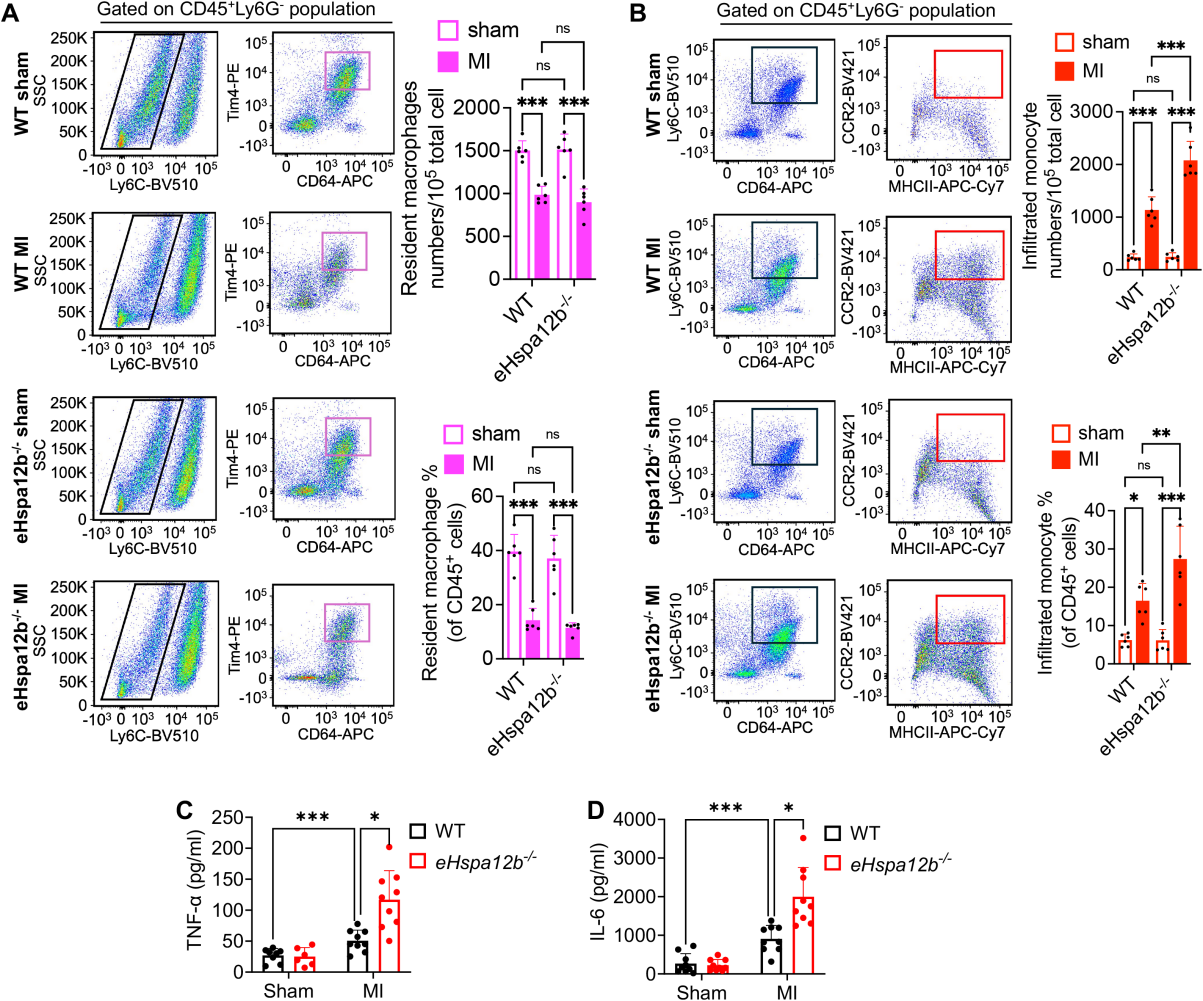
Endothelial *Hspa12b* deficiency enhances monocyte infiltration and reduces the resident macrophage population 3 days post-MI. WT and endothelial cell-specific *Hspa12b^-/-^* mice were subjected to MI or sham surgery. Heart tissues were collected 3 days following MI to evaluate the populations of cardiac-infiltrated monocyte and resident macrophages via flow cytometry. After removal of dead cells, immune cells were first gated as CD45^+^. **(A)** Cardiac resident macrophages were gated as CD45^+^ Ly6G^-^ Ly6C^-^ CD64^+^ Tim4^+^. N=6. **(B)** Cardiac infiltrated monocytes were gated as CD45^+^ Ly6G^-^ Ly6C^hi^ CD64^+^ CCR2^+^ MHC II^+^. N=6. **(C, D)** The inflammatory cytokine TNF-α and IL-6 production in mouse plasma was measured by ELISA. N=6-9. Comparisons of data between groups were made using two-way ANOVA followed by Tukey’s procedure. *P < 0.05, **P < 0.01, ***P < 0.001 compared with the indicated groups. ns, no significance.

To define the phenotypes of accumulated macrophages in the myocardium after MI, we collected heart tissues 3 days post-MI to evaluate the numbers of resident and infiltrated macrophages. The grating strategy and fluorescence minus one (FMO) controls used to identify cardiac macrophages and monocytes by flow cytometry are shown in **Supplementary Figures S2**, **S3**, respectively. Immune cells were initially gated as CD45^+^, followed by exclusion of neutrophils (Ly6G^+^). CD64, an evolutionarily conserved receptor, is exclusively expressed on monocytes and macrophages in both mice and humans, making it a reliable general marker for identifying cardiac macrophages and monocytes (5, 6). We observed that the resident macrophage (CD45^+^ Ly6G^-^ Ly6C^-^ CD64^+^ Tim4^+^) numbers were decreased (**Figure 2A**), while the infiltrated monocytes (CD45^+^ Ly6G^-^ Ly6C^hi^ CD64^+^ CCR2^+^ MHC II^+^) numbers were increased (**Figure 2B**), in both WT and *Hspa12b* ^-/-^ mice 3 days following MI. It is acknowledged that resident macrophages are replaced by infiltrated monocytes in the acute cardiac injury following MI (9, 30, 31), which is consistent with our observation. Interestingly, our data show that the number of infiltrated monocytes (red-boxed populations) was significantly greater in *Hspa12b* ^-/-^ mice than in WT mice following MI (**Figure 2B**). In contrast, no significant difference was observed in resident macrophage counts (magenta-boxed populations) between WT and *Hspa12b* ^-/-^ mice following MI (**Figure 2A**). Previous studies have reported that infiltration of CCR2^+^ monocytes into the myocardium is a maladaptive response because it promotes cardiac inflammation, increased oxidative stress and cardiac fibrosis (32, 33). In contrast, cardiac resident macrophages play a critical role in preserving cardiac function by enhancing angiogenesis (34). We then measured inflammatory cytokine levels and found that production of TNFα and IL-6 was significantly increased in the plasma following MI (**Figures 2C, D**), suggesting the activation of immune response. In addition, deficiency of Hspa12b strongly elevated the pro-inflammatory cytokine (TNFα and IL-6) production (**Figures 2C, D**), which is consistent with our observation that Hspa12b deficiency promoted infiltrated (pro-inflammatory) monocytes following MI (**Figure 2B**). Together, our data reveal that deficiency of HSPA12B in endothelial cells stimulates the infiltration of pro-inflammatory monocytes, potentially resulting in cardiac maladaptation following MI injury.

### Endothelial cell HSPA12B regulates macrophage phenotypes following hypoxic challenge

**Figure 3:**
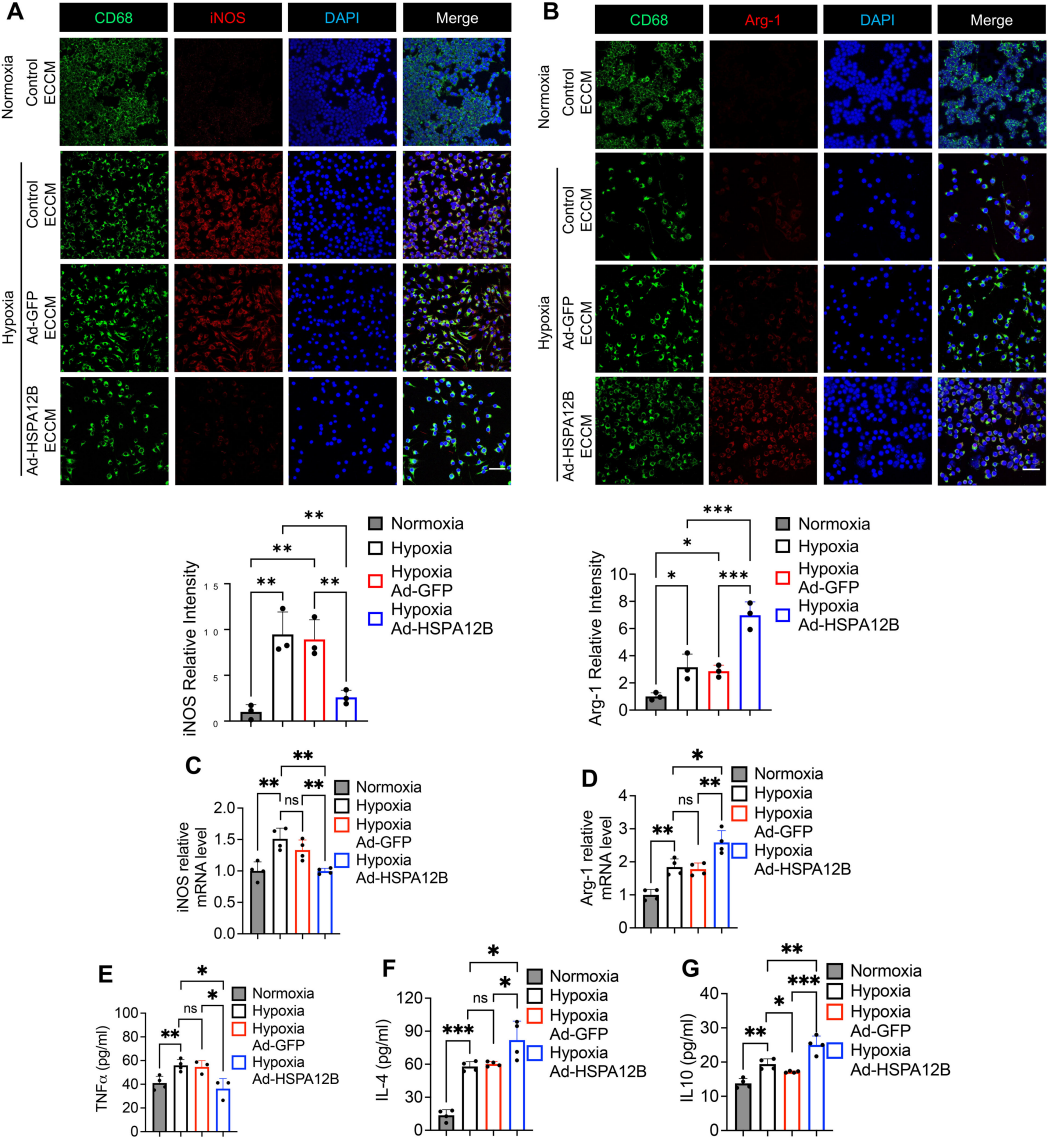
Endothelial cell HSPA12B regulates macrophage phenotypes and cytokine profiles following hypoxic challenge. HUVECs were transfected with adenovirus expressing HSPA12B (Ad-HSPA12B) or Ad-GFP (vector control) 24 hours before being subjected to hypoxia. Macrophages (RAW264.7 cells) were then incubated with the supernatants of endothelial-conditioned medium (ECCM) for 6 hours. **(A)** Macrophages were stained with anti-CD68 and anti-iNOS antibodies to evaluate M1 pro-inflammatory phenotype polarization. N=3. Scale bar: 50µm. **(B)** Macrophages were stained with anti-CD68 and anti-Arg-1 antibodies to evaluate M2 pro-regenerative phenotype polarization. N=3. Scale bar: 50µm. **(C, D)** The mRNA levels of *iNOS* and *Arg-1* in macrophages (RAW264.7 cells) were measured via qRT–PCR (N=4). **(E-G)** The production of the inflammatory cytokines TNF-α, IL-4 and IL-10 in macrophages (RAW264.7 cells) was measured via ELISA (N=3-5). Comparisons of data between groups were made using one-way ANOVA followed by Tukey’s test. *P < 0.05, **P < 0.01, ***P < 0.001 compared with the indicated groups. ns, no significance.

**Figure 4:**
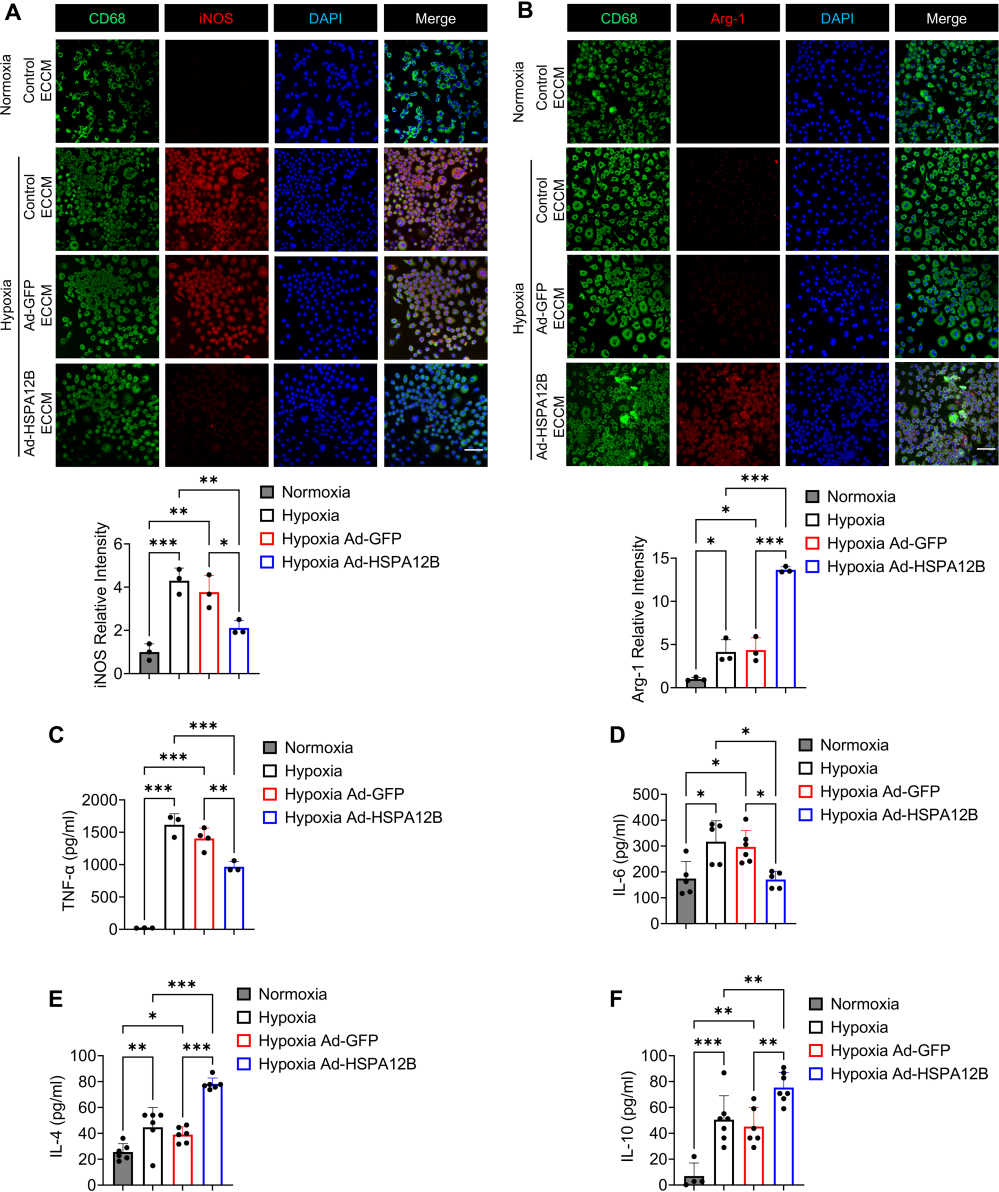
Endothelial cell HSPA12B regulates bone marrow-derived macrophage (BMDM) phenotypes following hypoxic challenge. HUVECs were transfected with adenovirus expressing HSPA12B (Ad-HSPA12B) or Ad-GFP 24 hours before being subjected to hypoxia. Bone marrow-derived macrophages were then incubated with the supernatants of endothelial-conditioned medium for 6 hours. **(A)** Macrophages were stained with anti-CD68 and anti-iNOS antibodies to evaluate M1 pro-inflammatory phenotype polarization. N=3. Scale bar: 50µm. **(B)** Macrophages were stained with anti-CD68 and anti-Arg-1 antibodies to evaluate M2 pro-regenerative phenotype polarization. N=3. Scale bar: 50µm. **(C-F)** The production of the inflammatory cytokines TNF-α, IL-6, IL-4 and IL-10 in macrophages was measured via ELISA (N=3-7). Comparisons of data between groups were made using one-way ANOVA followed by Tukey’s test. *P < 0.05, **P < 0.01, ***P < 0.001 compared with the indicated groups.

We then conducted *in vitro* experiments to elucidate how endothelial cell HSPA12B regulates macrophage phenotypes post-MI. Endothelial cells (HUVECs) were transfected with adenovirus expressing HSPA12B (Ad-HSPA12B) or Ad-GFP as a vector control 24 hours prior to hypoxia induction. The supernatants were collected as endothelial cell-conditioned medium (ECCM). We then incubated the macrophages with ECCM for 6 hours, followed by hypoxia challenge. The phenotypes of the macrophages were evaluated via immunofluorescence staining with anti-iNOS (M1, pro-inflammatory phenotype) and anti-Arg-1 (M2, pro-regenerative phenotype) antibodies. Our data revealed that hypoxia induced iNOS expression in macrophages when compared to normoxia conditions (**Figures 3A**, **4C**). However, ECCM from Ad-HSPA12B-transduced endothelial cells suppressed iNOS expression in macrophages following hypoxia (**Figures 3A**, **4C**). In addition, ECCM from Ad-HSPA12B-transduced endothelial cells enhanced the expression of Arg-1 in the macrophages under hypoxia conditions (**Figures 3B**, **4D**). Consistently, the effects of ECCM from Ad-HSPA12B-transduced endothelial cells on macrophage polarization were observed in BMDMs (**Figures 4A, B**). Taken together, our data demonstrated that hypoxia promotes a macrophage activation and that endothelial cell HSPA12B primes macrophages toward a pro-regenerative phenotype following hypoxic challenge.

### Endothelial cell HSPA12B regulates macrophage cytokine profiles following hypoxic challenge

To validate the role of endothelial HSPA12B in the regulation of macrophage activation, we examined the effect of ECCM on the cytokine profiles of macrophages in response to hypoxic challenge. Macrophages were incubated with ECCM that were generated from control, Ad-GFP-, or Ad-HSPA12B-transfected endothelial cells, followed by hypoxic challenge as described above. The levels of cytokines in the supernatants were measured with commercially available ELISA kits. **Figure 3E** shows that Ad-HSPA12B ECCM significantly attenuated the levels of pro-inflammatory cytokine TNF-α in macrophages compared with those in macrophages incubated with control ECCM or ECCM from Ad-GFP transfected endothelial cells following hypoxic challenge. In contrast, the levels of IL-4 and IL-10, which are associated with the pro-regenerative phenotype (35), were increased in macrophages treated with Ad-HSPA12B ECCM (**Figures 3F, G**). In addition, we validated our observations using BMDMs. As shown in **Figures 4C-F**, production of pro-inflammatory cytokines (TNF-α and IL-6) was decreased, while pro-regenerative cytokines (IL-4 and IL-10) was increased, in BMDMs incubated with Ad-HSPA12B ECCM, when compared to those incubated with control ECCM or ECCM from Ad-GFP transfected endothelial cells following hypoxic challenge. These data suggest that endothelial cell HSPA12B regulates macrophage polarization toward a pro-regenerative phenotype in response to hypoxic challenge.

### Endothelial cell HSPA12B can be transmitted into macrophages via endothelial cell exosome secretion

**Figure 5:**
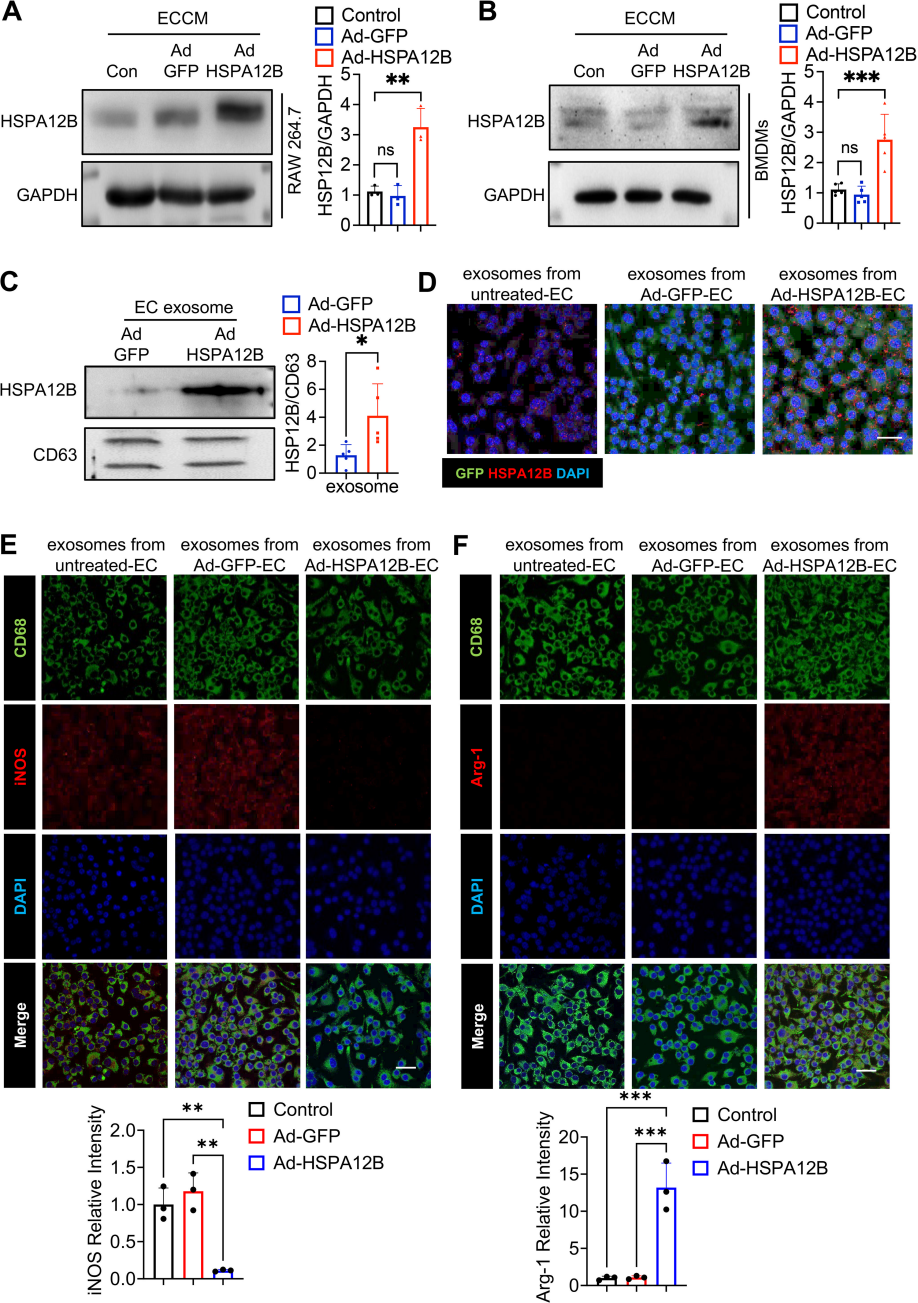
Endothelial cell HSPA12B can be taken up by macrophages via endothelial cell exosomes. HUVECs were transfected with Ad-HSPA12B or Ad-GFP 24 hours before they were subjected to hypoxia. Macrophages (RAW264.7 cells) (**A**, N=3) and bone marrow-derived macrophages (BMDMs) (**B**, N=5) were then incubated with ECCM for 6 hours. HSPA12B protein levels were measured via Western blotting. **(C)** Exosomes were isolated from endothelial cell-conditioned medium, and exosomal HSPA12B was measured by Western blotting (N=5). **(D)** BMDMs were treated with exosomes derived from untreated endothelial cells, Ad-GFP-transfected endothelial cells or Ad-HSPA12B-transfected endothelial cells. The expression of HSPA12B in BMDMs was measured via immunofluorescence staining (N=3). Scale bar: 50µm. **(E)** BMDMs were incubated with endothelial exosomes and stained with anti-CD68 and anti-iNOS antibodies. **(F)** BMDMs were incubated with endothelial exosomes and stained with anti-CD68 and anti-Arg-1 antibodies (N=3). Scale bar: 50µm. Comparisons of data between groups were made using one-way ANOVA followed by Tukey’s test. *P < 0.05, **P < 0.01, ***P < 0.001 compared with the indicated groups.

To determine how endothelial cell HSPA12B regulates macrophage phenotypes, we examined whether endothelial cell HSPA12B can be transmitted into macrophages. We incubated the macrophages with ECCM and then subjected them to hypoxia for 6 hours. We then harvested the cells and examined whether HSPA12B was present in the macrophages. As shown in **Figure 5A**, macrophages that were incubated with the ECCM generated from Ad-HSPA12B-transfected endothelial cells presented increased protein levels of HSPA12B. Similar results were also observed in BMDMs (**Figure 5B**), indicating that endothelial cell HSPA12B can be transmitted into macrophages.

Exosomes play important roles in mediating cell–cell or organ–organ communication (36). We examined whether HSPA12B is released via exosome secretion and whether exosomal HSPA12B is taken up by macrophages. We isolated exosomes from ECCM and examined the HSPA12B levels in the isolated exosomes. **Figure 5C** shows that the exosomes isolated from ECCM generated from Ad-HSPA12B-transfected endothelial cells contained higher levels of HSPA12B, when compared with the exosomes isolated from the ECCM of Ad-GFP-transfected endothelial cells. These data suggest that endothelial HSPA12B can be released via exosome secretion. To examine whether endothelial exosomes can be taken up by macrophages, we incubated macrophages (BMDMs) with isolated endothelial exosomes and examined the levels of HSPA12B in the macrophages. **Figure 5D** shows that endothelial HSPA12B appears in macrophages treated with exosomes from either untreated endothelial cells or Ad-GFP-transfected endothelial cells. Interestingly, the levels of HSPA12B were markedly higher in macrophages treated with exosomes isolated from Ad-HSPA12B-transfected endothelial cells (**Figure 5D**). These data demonstrated that the HSPA12B protein can be released by endothelial cells via exosome secretion and is subsequently transmitted into macrophages.

### Endothelial exosomal HSPA12B regulates macrophage polarization toward the M2 phenotype

To confirm the role of exosomal eHSPA12B in ECCM-induced changes in macrophage phenotypes and cytokine expression (**Figures 3**, **4**), we isolated exosomes from ECCM as described above. Subsequently, we incubated BMDMs with endothelial exosomes followed by hypoxia for 6 hours. iNOS and Arg-1 in macrophages were examined via immunofluorescence staining. As shown in **Figure 5E**, hypoxia led to an increase in iNOS staining in macrophages treated with exosomes derived from control ECCM and Ad-GFP ECCM, but not in those derived from Ad-HSPA12B ECCM. Conversely, incubation of macrophages with exosomal HSPA12B increased Arg-1 staining compared with incubation with exosomes isolated from control or Ad-GFP-transfected endothelial cells (**Figure 5F**). These data indicate that HSPA12B-containing exosomes can regulate the macrophage phenotype after hypoxic stimulation.

### Endothelial HSPA12B suppresses TLR4/MyD88 signaling in macrophages after hypoxia

**Figure 6:**
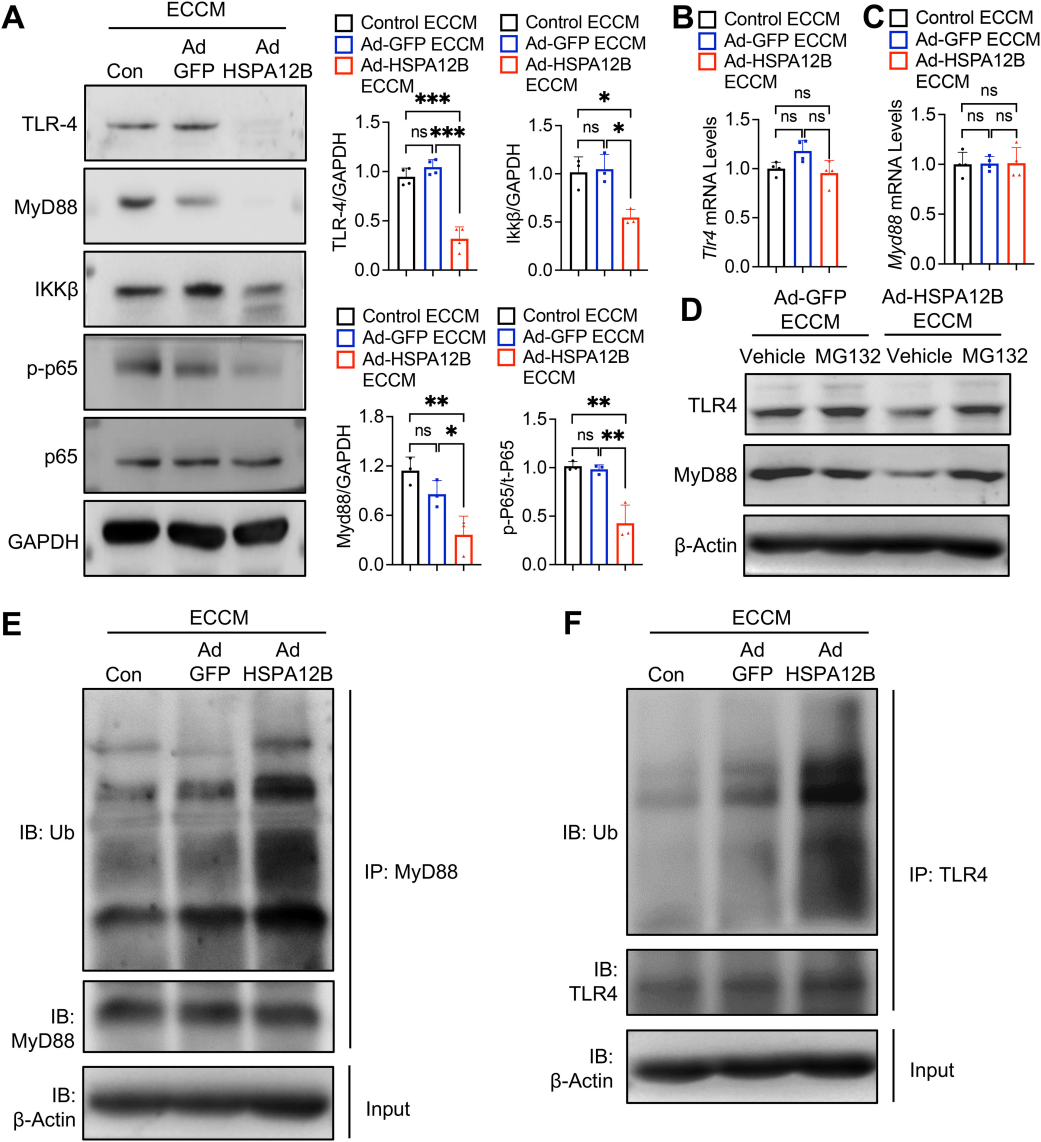
Endothelial HSPA12B suppresses TLR4/MyD88 signaling in macrophages after hypoxia. HUVECs were transfected with Ad-HSPA12B or Ad-GFP 24 hours before they were subjected to hypoxia. The RAW264.7 macrophages were then incubated with the supernatants of ECCM for 6 hours. **(A)** The expression of TLR4, MyD88, IKKβ and phosphorylated NF-κB was measured via Western blotting (N=3-4). **(B, C)** The mRNA levels of *Tlr4* and *Myd88* were measured by qRT-PCR (N=4). Macrophages were treated with the proteasome inhibitor MG132 in the presence of Ad-GFP or Ad-HSPA12B ECCMs. **(D)** The expression of TLR4 and MyD88 was measured via Western blotting. **(E, F)** The ubiquitination of TLR4 and MyD88 was measured by immunoprecipitation. Comparisons of data between groups were made via one-way ANOVA followed by Tukey’s test. *P < 0.05, **P < 0.01, ***P < 0.001 compared with the indicated groups. ns, no significance.

To investigate the mechanisms by which endothelial cell HSPA12B regulates the macrophage inflammatory response during hypoxia, we focused on its effects on TLR4-mediated NF-κB activation in macrophages (37). Macrophages were incubated with ECCM followed by hypoxic challenge for six hours. The cells were harvested, and the levels of TLR4, IKKβ and NF-κB subunit p65 were measured by Western blotting. **Figure 6A** shows that ECCM generated from Ad-HSPA12B-transfected endothelial cells significantly suppressed the protein expression of TLR4, MyD88 and IKK-β in hypoxic macrophages compared to macrophages treated with ECCM from untreated and Ad-GFP-transfected endothelial cells. These data indicate that endothelial cell HSPA12B regulates TLR4-mediated NF-κB activation, which plays an important role in pro-inflammatory cytokine production in macrophages.

To elucidate the mechanisms by which HSPA12B regulates TLR4/IKKβ/NF-κB activation, we first examined the mRNA levels of *TLR4* and *MyD88* in macrophages. We observed that Ad-HSPA12B ECCM did not alter the mRNA expression of *TLR4* and *MyD88* in macrophages (**Figures 6B, C**). Next, we examined whether HSPA12B containing ECCM affects TLR4 and MyD88 ubiquitination and degradation, thereby regulating TLR4/IKKβ/NF-κB activation. We treated macrophages with the proteasome inhibitor MG132 in the presence of Ad-GFP or Ad-HSPA12B ECCMs and examined the TLR4/MyD88 levels. MG132 is a potent and reversible proteasome inhibitor that blocks the degradation of ubiquitin-conjugated proteins (38). As shown in **Figure 6D**, inhibition of protein degradation by MG132 reversed HSPA12B suppressed TLR4 and MyD88 expression in macrophages. In addition, we detected increased ubiquitination of TLR4 and MyD88 in macrophages treated with Ad-HSPA12B ECCM (**Figures 6E, F**). Together, our data demonstrate that endothelial cell HSPA12B can induce the ubiquitination and degradation of TLR4 and MyD88 in macrophages, which attenuates macrophage pro-inflammatory responses following hypoxia.

## Discussion

In this study, we demonstrated that endothelial cell-specific *Hspa12b* exerts a novel role in mediating crosstalk between endothelial cells and cardiac macrophages during MI. Our findings reveal a previously unknown function of endothelial cell HSPA12B in the regulation of the cardiac macrophage population and phenotype within the myocardium following MI. Mechanistic studies revealed that HSPA12B is released from endothelial cells via exosome secretion and is subsequently transmitted into macrophages to suppress TLR4/MyD88/NF-κB activation.

Han and colleagues first discovered that HSPA12B is expressed in atherosclerotic lesions and then identified it as an endothelium-specific chaperone required for angiogenesis (18, 19). Our previous investigations suggested that deficiency of endothelial cell-specific *Hspa12b* exacerbates cardiac dysfunction post-MI by regulating cardiac angiogenesis (20). In contrast, the overexpression of HSPA12B protects against cerebral ischemic injury through the activation of PI3K/Akt signaling (39). During the initial stage of MI, macrophages accumulate in the infarcted heart to regulate inflammatory responses, cardiac damage, cardiac repair, and angiogenesis (4, 40). Our results show that, compared with the sham control, MI promotes the recruitment of monocytes into the myocardium. Importantly, endothelial *Hspa12b* depletion further induced monocyte infiltration in the myocardium, which was accompanied by worsening cardiac dysfunction following MI. To eliminate the possibility that reduced cardiac function is due to variability in infarct size or area-at-risk rather than *eHspa12b* deficiency, we ensured consistent surgical technique, including identical LAD ligation location and duration, across all experimental groups. In addition, we performed TTC staining to evaluate infarct size following MI. While we did not employ a counterstain such as Evans blue dye or fluorescent microspheres to define the area-at-risk, this approach aligns with standard practices in myocardial infarction models where infarct size comparisons are made under tightly controlled and consistent surgical conditions. TTC staining shows no significant differences in the infarcted myocardium aera between WT and *eHspa12b* deficient mice following MI, supporting the conclusion that the exacerbated cardiac dysfunction is attributable to *eHspa12b* deficiency.

Next, we defined the phenotype of the macrophages that were recruited into the heart. During the inflammatory phase of infarct repair, Ly6C^hi^ monocytes and macrophages are recruited and expanded, which is accompanied by a decrease in resident macrophages (9, 41, 42). We observed that endothelial cell *Hspa12b* deficiency further increased the infiltrated monocyte population, while has no significant impacts on the number of resident macrophages, in MI hearts, suggesting the important role of endothelial HSPA12B in regulating immune response in MI hearts.

Previous evidence has demonstrated that endothelial cells play an essential regulatory role in macrophages following ischemic injury (13, 16, 17). A recent study indicated that endothelial cell activation induces M2-like macrophage polarization during muscle regeneration from ischemia (43). In the cardiovascular field, Kuang et al. revealed that the inhibition of endothelial-specific sphingosine 1-phosphate receptor 1 (S1pr1) downregulated the recruitment of reparative Ly-6C^low^ macrophages, resulting in exacerbated cardiac dysfunction and impaired angiogenesis post-MI (17). Further study showed that endothelial S1PR1 promotes macrophage proliferation via direct contact between endothelial cells and macrophages, not via conditioned medium from endothelial cells. In our study, we observed that endothelial cell-conditioned medium (ECCM) plays an important role in mediating the interaction between endothelial cells and macrophages during MI. Specifically, hypoxia stimulates macrophages to adopt a pro-inflammatory phenotype, and Ad-HSPA12B ECCM can convert macrophages toward a pro-regenerative phenotype after hypoxia. In addition, the overexpression of HSPA12B significantly ameliorated pro-inflammatory cytokine expression, while upregulated the production of pro-regenerative cytokines in macrophages after hypoxia. To further investigate how endothelial cell HSPA12B regulates the macrophage phenotype, we examined HSPA12B expression in macrophages. Surprisingly, our data revealed that endothelial HSPA12B is transmitted into macrophages. We and others have demonstrated the critical role of exosomes in cell–cell communication as vehicles for transferring proteins (23, 25, 36). Exosomes are circular membrane fragments released from the endosomal compartment of most cell types (36). Accumulating evidence has demonstrated that exosomes act as protein carriers for therapeutic cargo delivery (44, 45). We confirmed the presence of HSPA12B in exosomes isolated from Ad-HSPA12B-treated ECCM. Moreover, treatment of macrophages with endothelial exosomal HSPA12B promoted macrophage polarization toward a pro-regenerative phenotype, indicating that endothelial exosomal HSPA12B regulates macrophage polarization from a hypoxia-induced pro-inflammatory phenotype to a pro-regenerative phenotype.

Next, we explored the mechanisms by which endothelial cell HSPA12B regulates the macrophage inflammatory response. TLR4/MyD88-mediated NF-κB activation has been reported to facilitate M1 macrophage polarization in different diseases (46, 47). Therefore, we focused on whether endothelial exosomal HSPA12B could impact macrophage TLR4-mediated NF-κB activation during hypoxic challenge. Interestingly, we observed that exosomal eHSPA12B inhibited TLR4, MyD88, IKKβ and phosphorylated NF-κB expression in macrophages, suggesting that endothelial HSPA12B attenuated the TLR4/NF-κB signaling pathway in macrophages. However, the levels of *Tlr4* and *Myd88* mRNAs were not altered in response to Ad-HSPA12B transfection. Ubiquitination is a form of post-translational modification in which ubiquitin is attached to a target protein (48). We found that Ad-HSPA12B ECCM accelerated the ubiquitination of TLR4 and MyD88 in macrophages, suggesting that HSPA12B promoted TLR4/MyD88 degradation and NK-κB inactivation, contributing to macrophage polarization toward an anti-inflammatory phenotype. In future studies, we will elucidate the mechanisms by which endothelial HSPA12B activates ubiquitination in macrophages during myocardial ischemic injury.

This study has certain limitations. While Tek-Cre is a well-established and widely accepted tool for targeting endothelial cells, it does not differentiate between blood and lymphatic endothelial populations (49, 50). However, given that blood endothelial cells are the predominant subtype involved in vascular responses to injury, the primary conclusions of this study remain well supported. In addition, although this work focused on macrophage responses, the acute time points assessed suggest that neutrophils, which are highly responsive to endothelial cues (51–53), may also contribute to the observed effects. Investigating their involvement in future studies could provide further insight and complement the current findings.

In summary, the present study revealed a previously unknown role of endothelial cells in regulating macrophage activation through exosomal HSPA12B secretion, thereby protecting against MI-induced cardiac dysfunction.

## Data Availability

The raw data supporting the conclusions of this article will be made available by the authors, without undue reservation.

## Supplementary material

Supplementary Figure 1 Infarct size of wild type and endothelial cell-specific *Hspa12b* knockout myocardial infarction mice. WT and endothelial cell-specific *Hspa12b* knockout (*Hspa12b^-/-^*) mice were subjected to MI surgery. TTC staining was performed to measure cardiac infarct size. N=3-4. Comparisons of data between groups were made using unpaired t-test.

Supplementary Figure 2 Flow cytometry gating strategies for cardiac macrophages.

Supplementary Figure 3 Fluorescence minus one (FMO) controls and full stain controls for flow cytometry gating strategy.
