# Supplementary material for: Endothelial HSPA12B regulates myocardial monocyte infiltration and inflammatory activity after myocardial infarction
Source: Front Immunol. 2025 May 15;16:1587898. doi: 10.3389/fimmu.2025.1587898 (PMC12119287; doi:10.3389/fimmu.2025.1587898)
Supplement: Supplementary Figure 1 — Infarct size of wild type and endothelial cell-specific Hspa12b knockout myocardial infarction mice. WT and endothelial cell-specific Hspa12b knockout (Hspa12b-/- ) mice were subjected to MI surgery. TTC staining was performed to measure cardiac infarct size. N=3-4. Comparisons of data between groups were made using unpaired t-test. [file DataSheet1.pdf]

frontiers

The International Society for the Advancement of Cytometry (ISAC) has highlighted the importance of including comprehensive methodological information to ensure data reproducibility and reliability. In line with this, Frontiers in Immunology now requires authors to submit a checklist for manuscripts that involve flow or mass cytometry. This checklist helps standardize the reporting process, improving the quality and transparency of published data. By doing so, we support scientific progress, making it easier for other researchers to replicate and validate experiments.

This form should be submitted with any manuscripts using flow or mass cytometry.

Sample/specimen/material description

☐ Total blood

☐ PBMCs

☑ Organ digests

Other____

Did the samples suffer any treatment before or after incubation with the antibodies?

☐ Drug ____________________________________

☐ Cell permeabilization ______________________

☐ Dye _____________________________________

☐ Propidium iodine

☑ Not applicable

Other _______________________________________

Instrument and antibodies

Name of the Cytometer__BD FACSfortessa____________________________

Antibodies and targets	Fluorochrome/ Metal	Catalog number/Company
e.g. anti-CD4	FITC	Cat. XXX/ XXX ltd.
anti-F4/80	Alexa Fluor® 647	565853

anti-CD11b	PE-Cy™7	552850
anti-CCR2	PE	150610
anti-Ly-6C	FITC	128006
anti-CD14	PerCP/Cyanine5.5	123314
anti-CD64	PE	139304
anti-Mertk	FITC	151504

Data analyses

1. Name of the software FlowJo
2. Reference gating strategy in the manuscript or supplementary material

Gating strategy in (eg Figure X) Figure 2
