# Supplementary figures and images for: Endothelial HSPA12B regulates myocardial monocyte infiltration and inflammatory activity after myocardial infarction
Source: Front Immunol. 2025 May 15;16:1587898. doi: 10.3389/fimmu.2025.1587898 (PMC12119287; doi:10.3389/fimmu.2025.1587898)

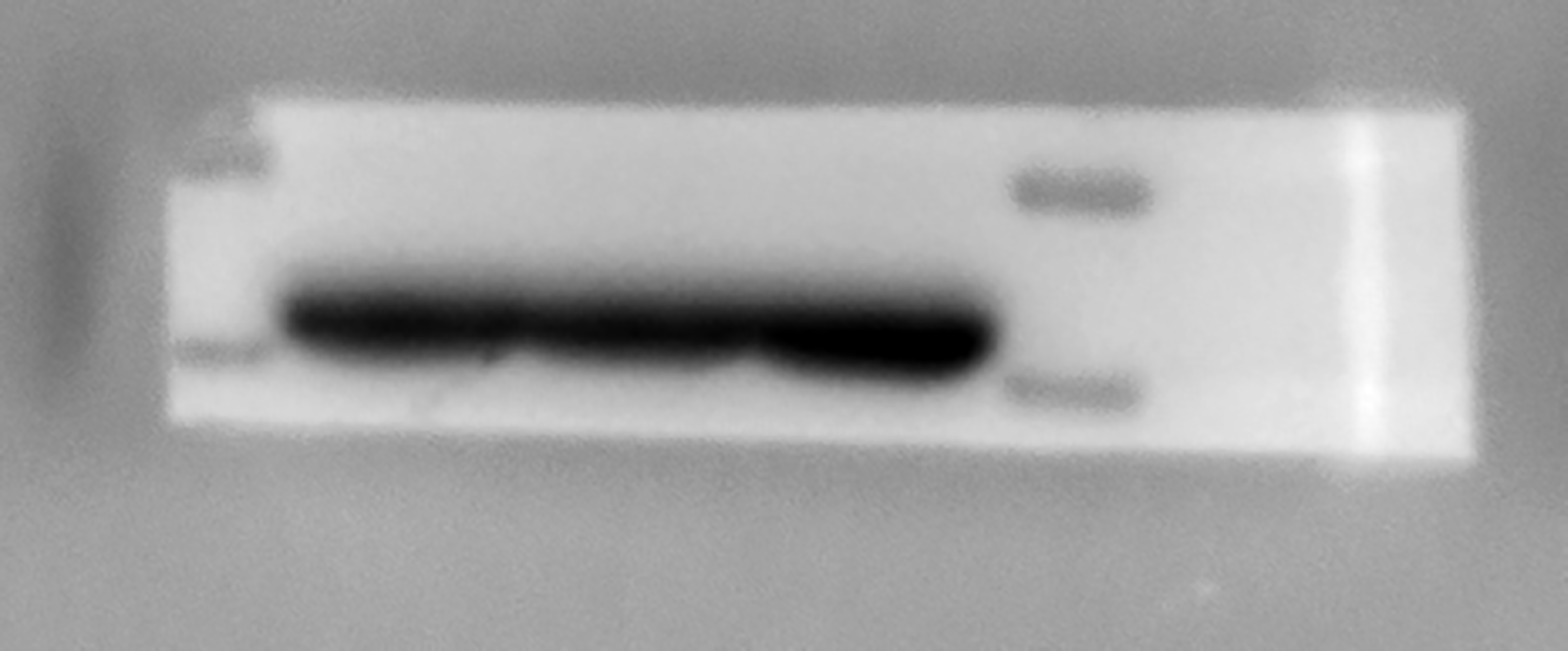

Supplement: Supplementary Figure 2 — Flow cytometry gating strategies for cardiac macrophages. [file DataSheet2.zip › uncropped and unedited western blot images (Part 1)/actin 1.tif]

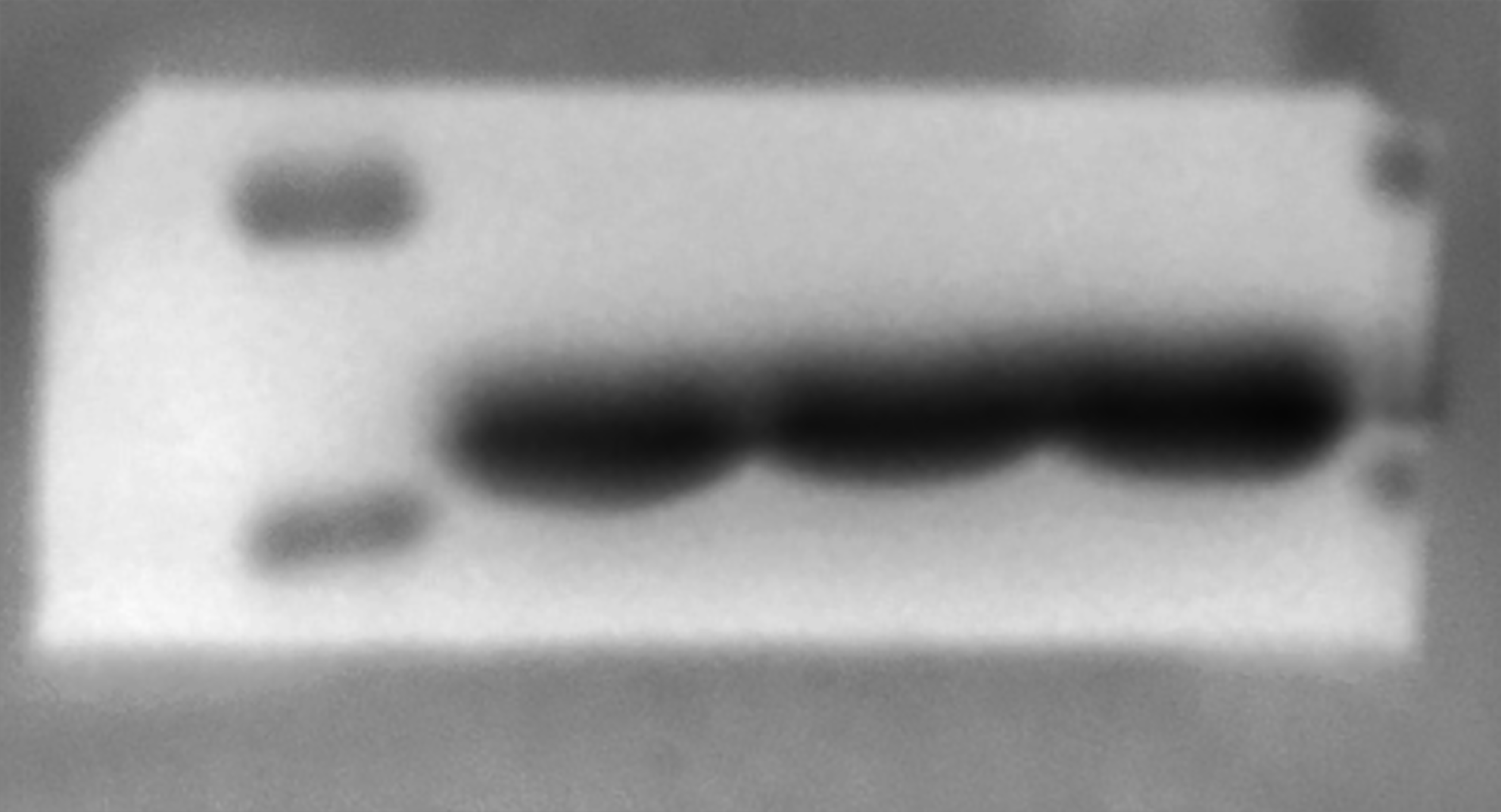

Supplement: Supplementary Figure 2 — Flow cytometry gating strategies for cardiac macrophages. [file DataSheet2.zip › uncropped and unedited western blot images (Part 1)/actin 2.tif]

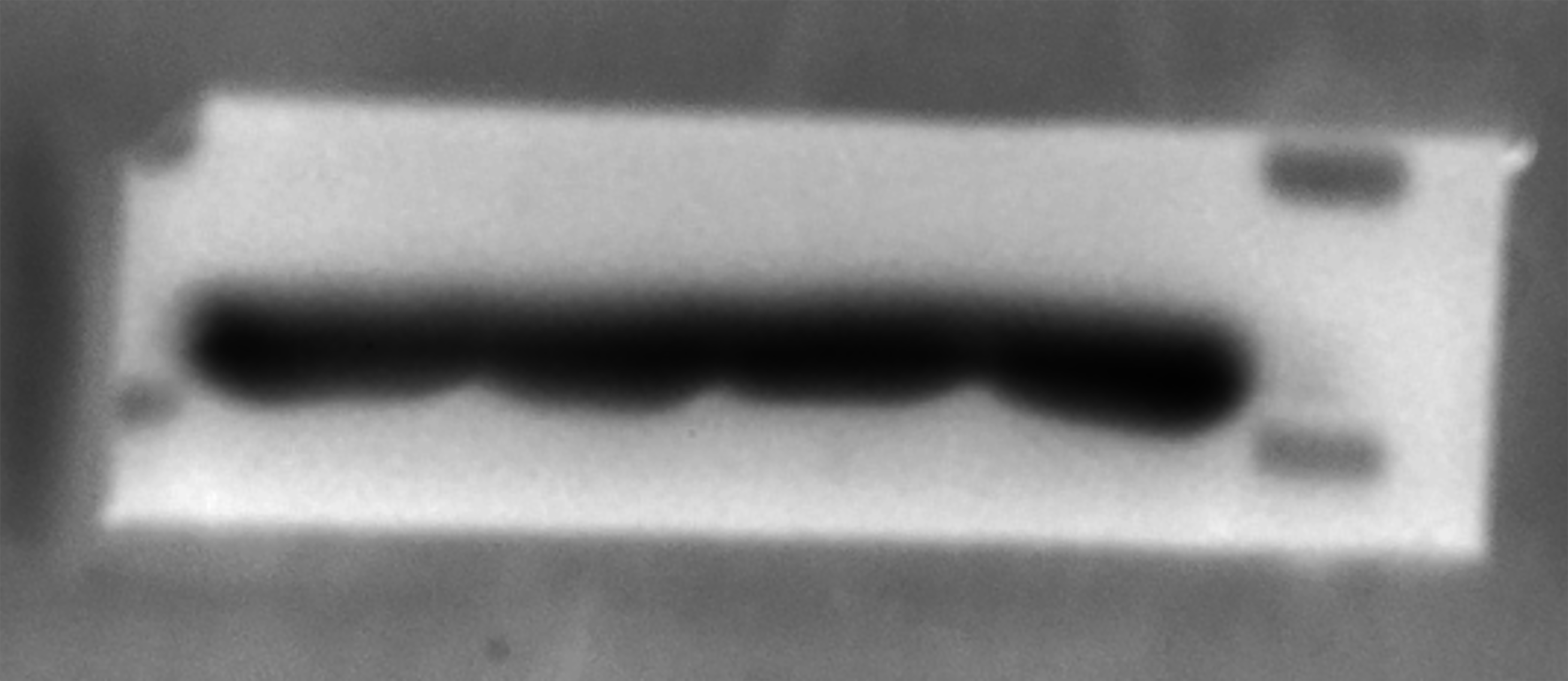

Supplement: Supplementary Figure 2 — Flow cytometry gating strategies for cardiac macrophages. [file DataSheet2.zip › uncropped and unedited western blot images (Part 1)/actin 3.tif]

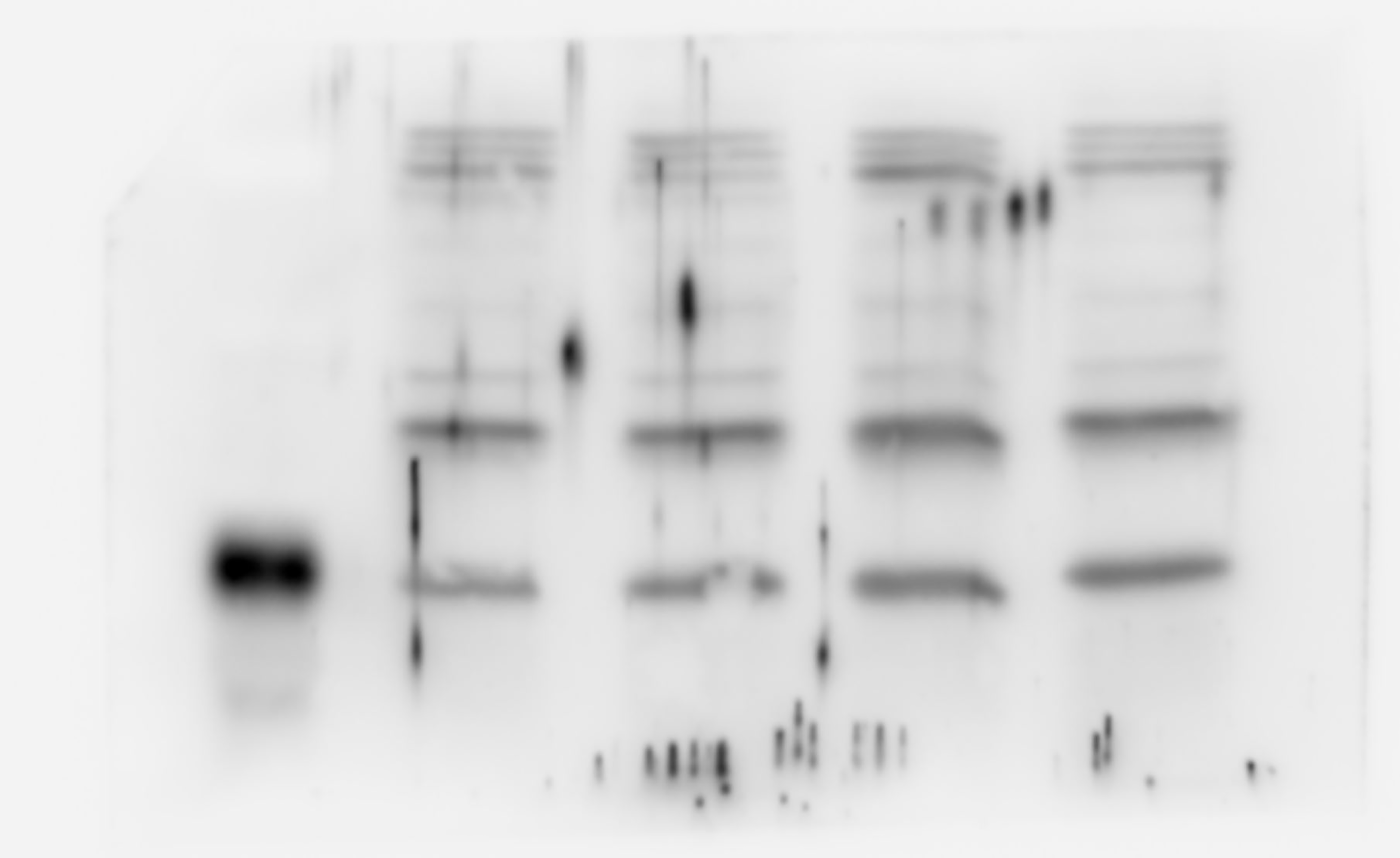

Supplement: Supplementary Figure 2 — Flow cytometry gating strategies for cardiac macrophages. [file DataSheet2.zip › uncropped and unedited western blot images (Part 1)/cd63.tif]

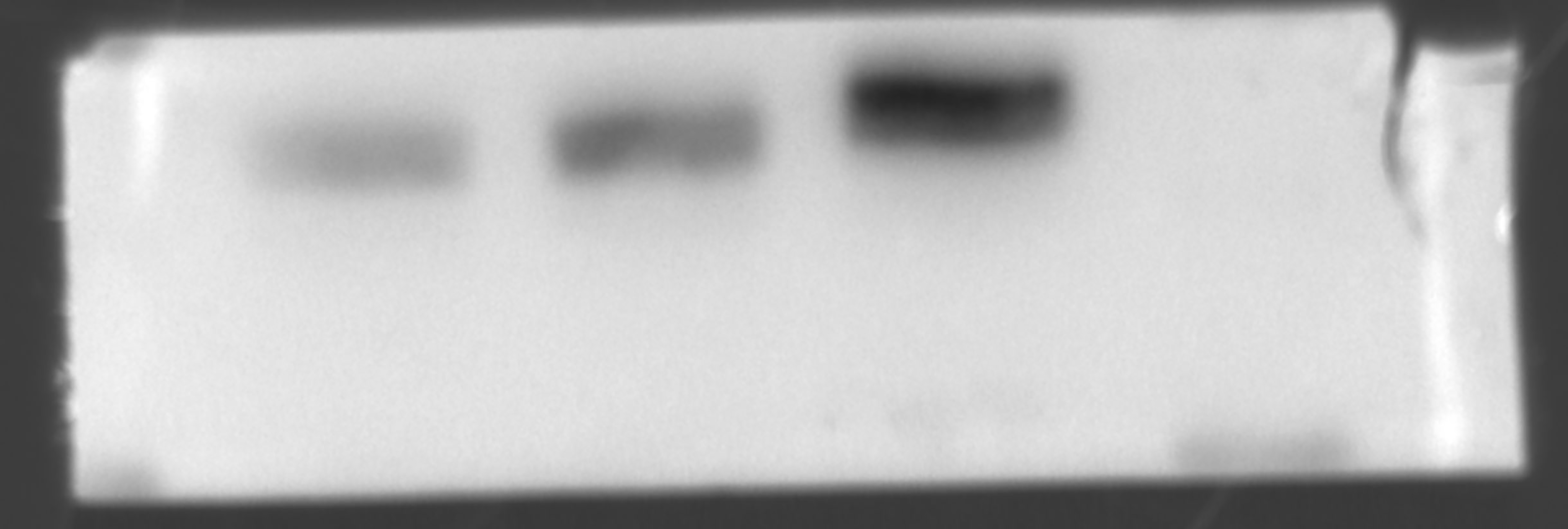

Supplement: Supplementary Figure 2 — Flow cytometry gating strategies for cardiac macrophages. [file DataSheet2.zip › uncropped and unedited western blot images (Part 1)/hspa12b 1.tif]

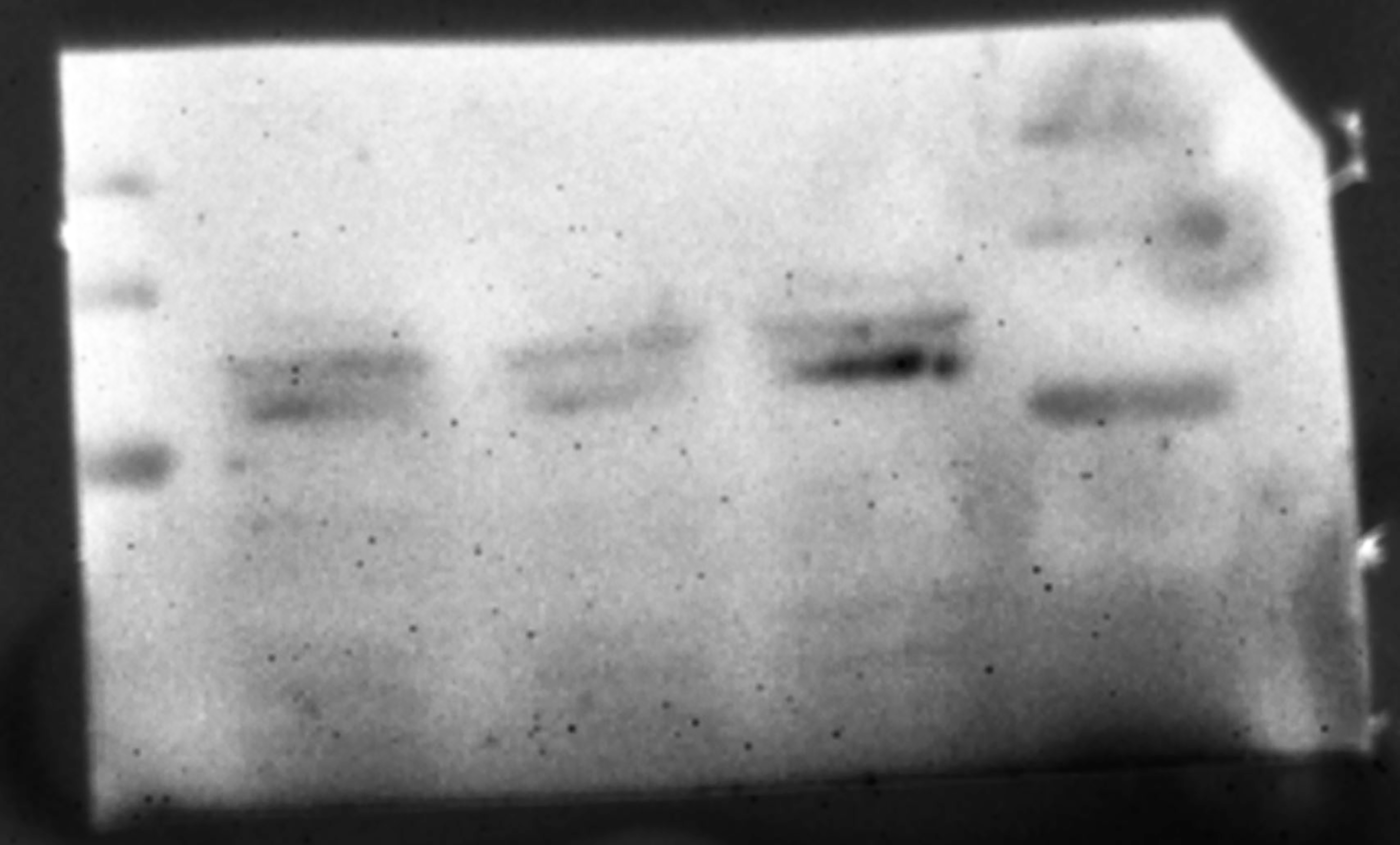

Supplement: Supplementary Figure 2 — Flow cytometry gating strategies for cardiac macrophages. [file DataSheet2.zip › uncropped and unedited western blot images (Part 1)/hspa12b 2.tif]

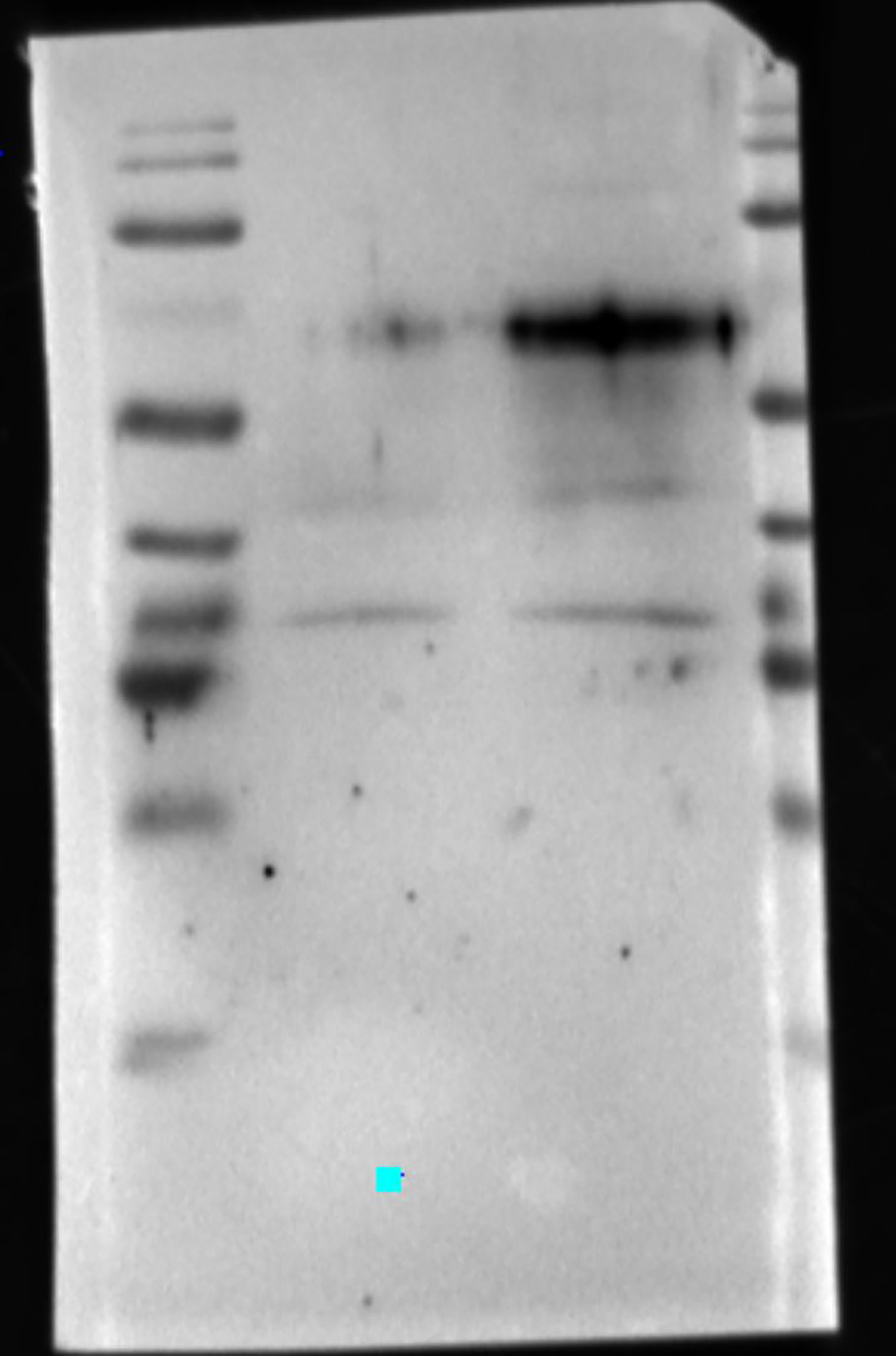

Supplement: Supplementary Figure 2 — Flow cytometry gating strategies for cardiac macrophages. [file DataSheet2.zip › uncropped and unedited western blot images (Part 1)/hspa12b exo.tif]

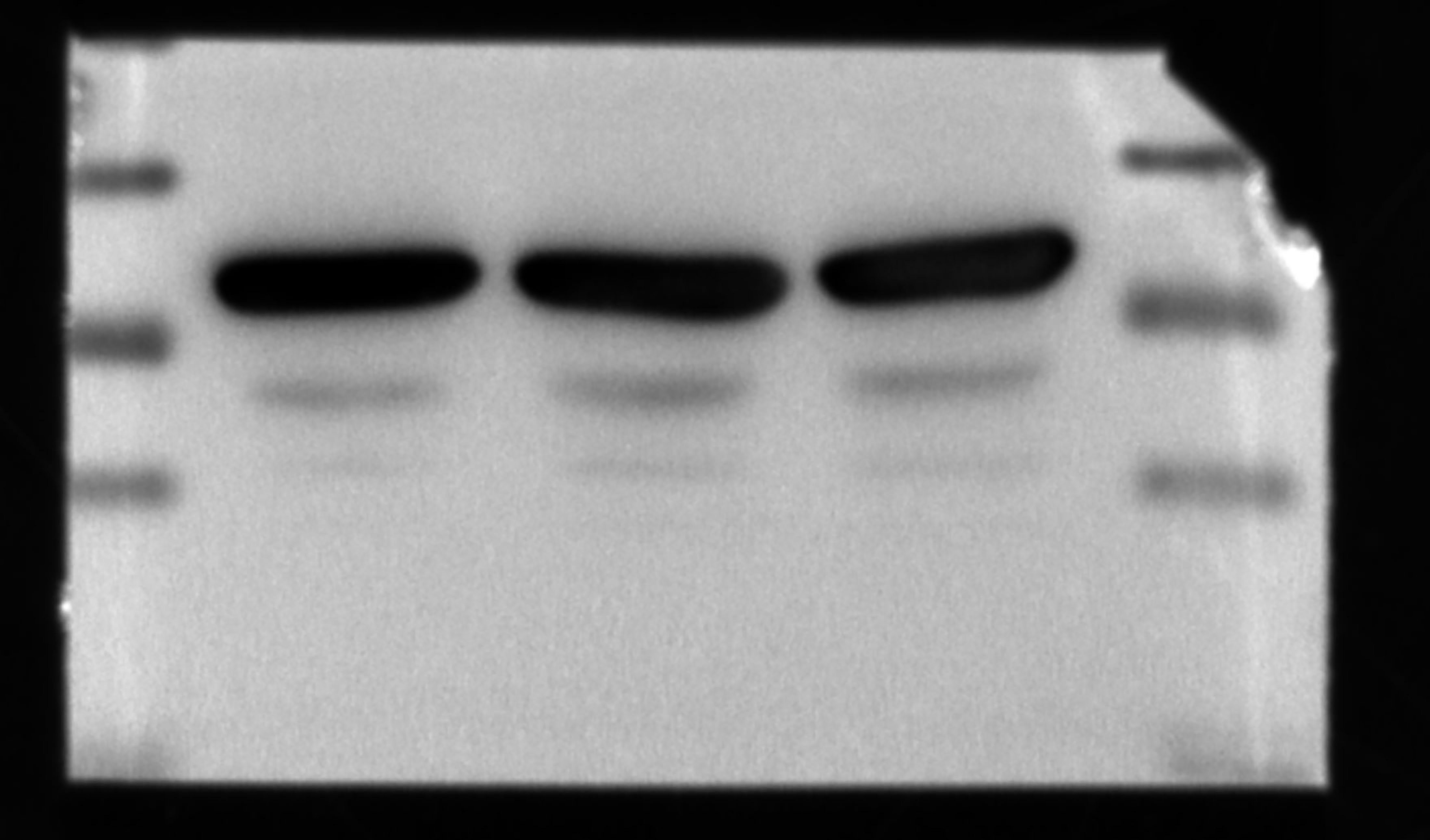

Supplement: Supplementary Figure 2 — Flow cytometry gating strategies for cardiac macrophages. [file DataSheet2.zip › uncropped and unedited western blot images (Part 1)/gapdh 1.tif]

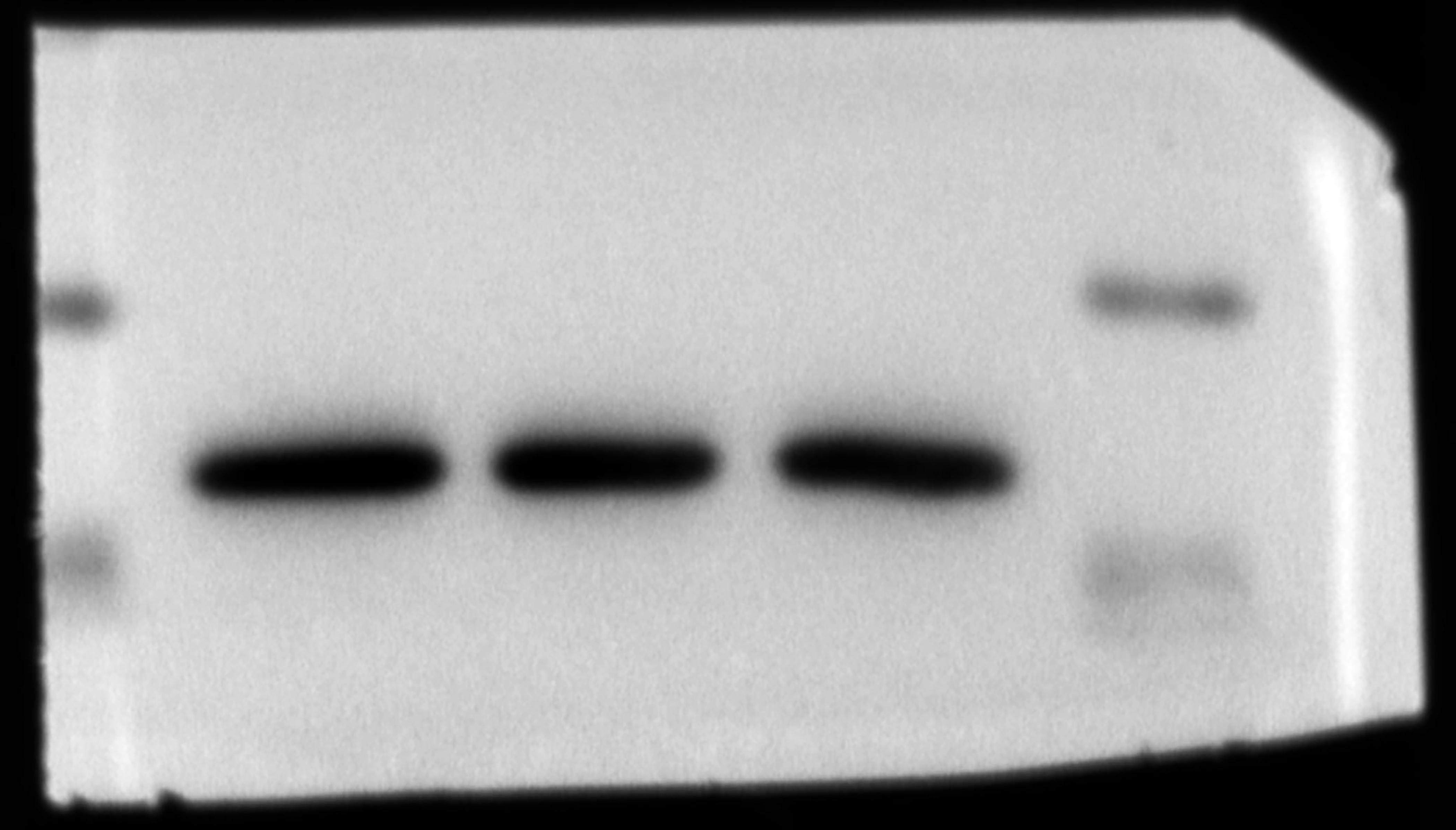

Supplement: Supplementary Figure 2 — Flow cytometry gating strategies for cardiac macrophages. [file DataSheet2.zip › uncropped and unedited western blot images (Part 1)/gapdh 3.tif]

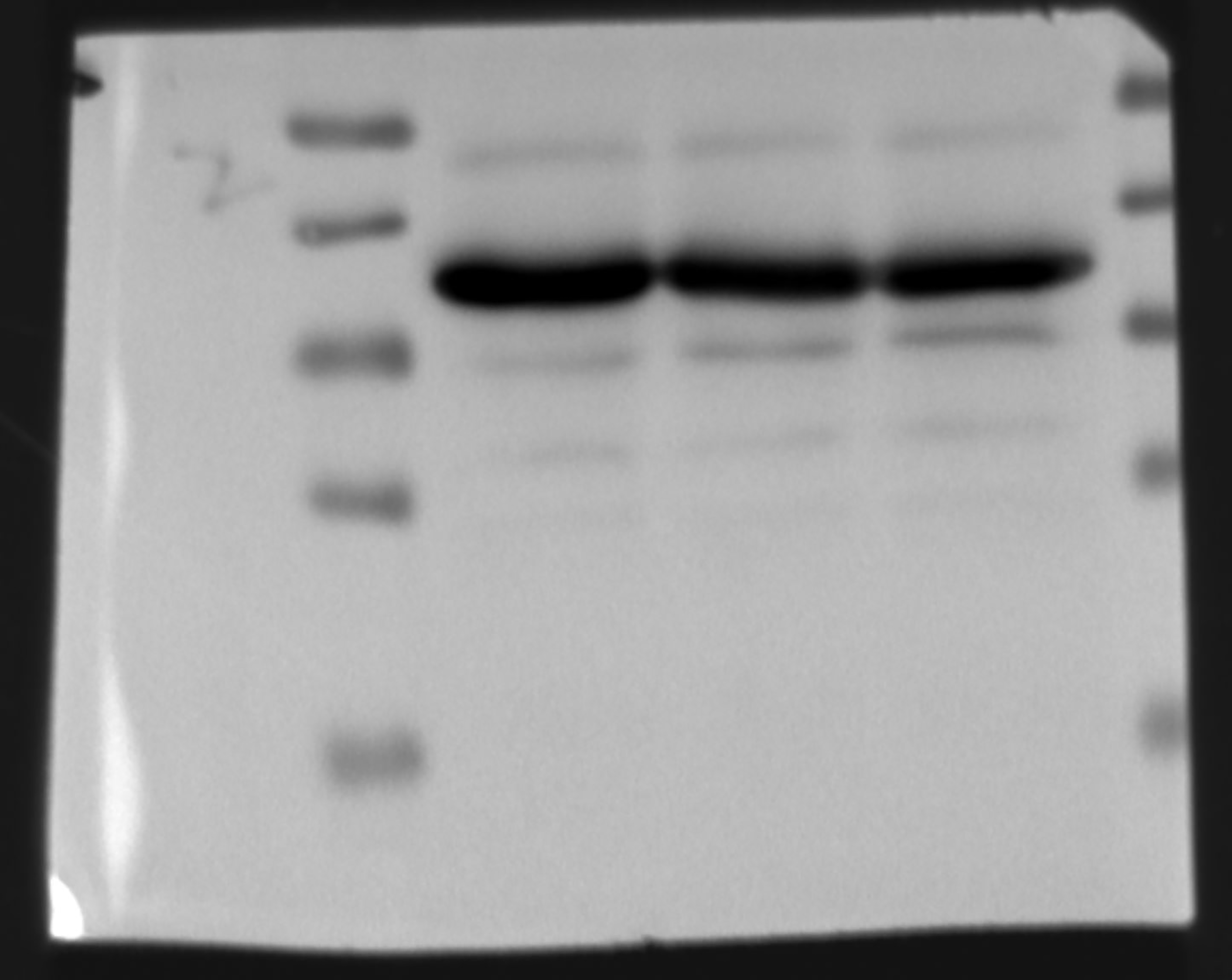

Supplement: Supplementary Figure 2 — Flow cytometry gating strategies for cardiac macrophages. [file DataSheet2.zip › uncropped and unedited western blot images (Part 1)/gapdh 2.tif]

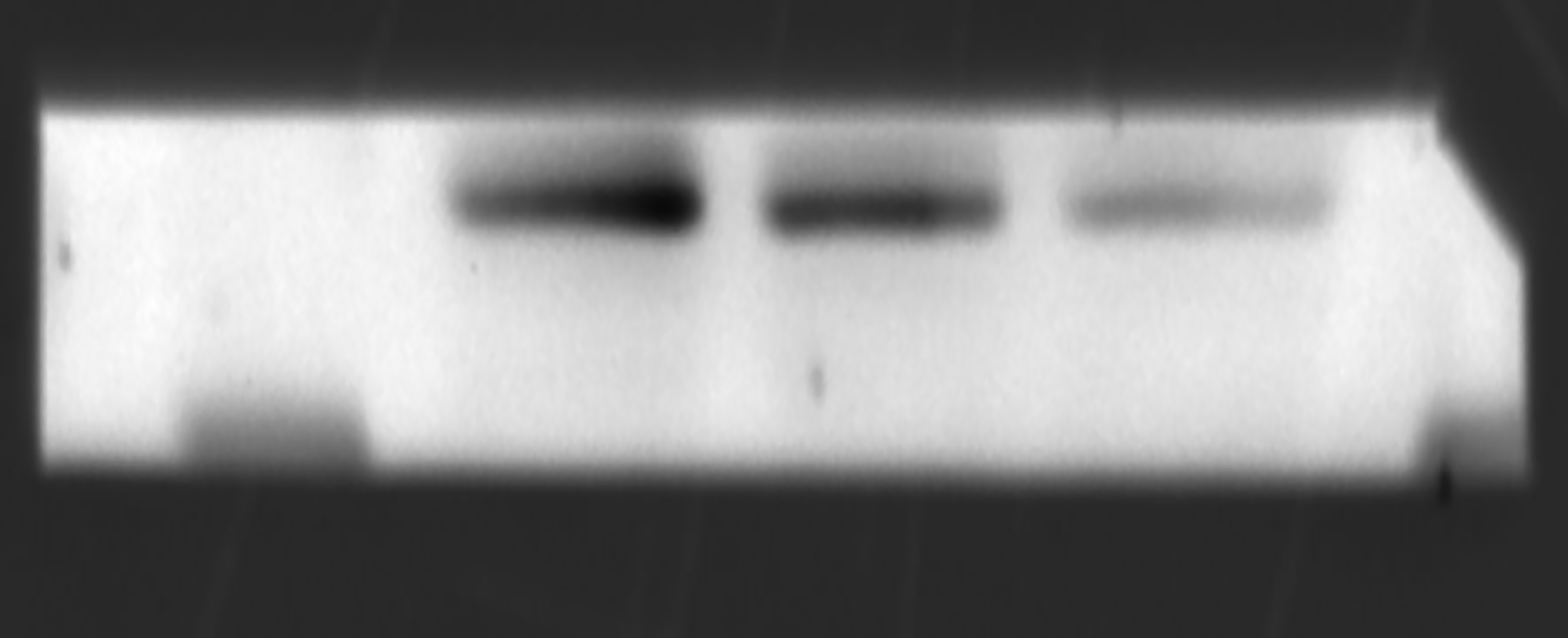

Supplement: Supplementary Figure 3 — Fluorescence minus one (FMO) controls and full stain controls for flow cytometry gating strategy. [file DataSheet3.zip › uncropped and unedited western blot images (Part 2)/pp65.tif]

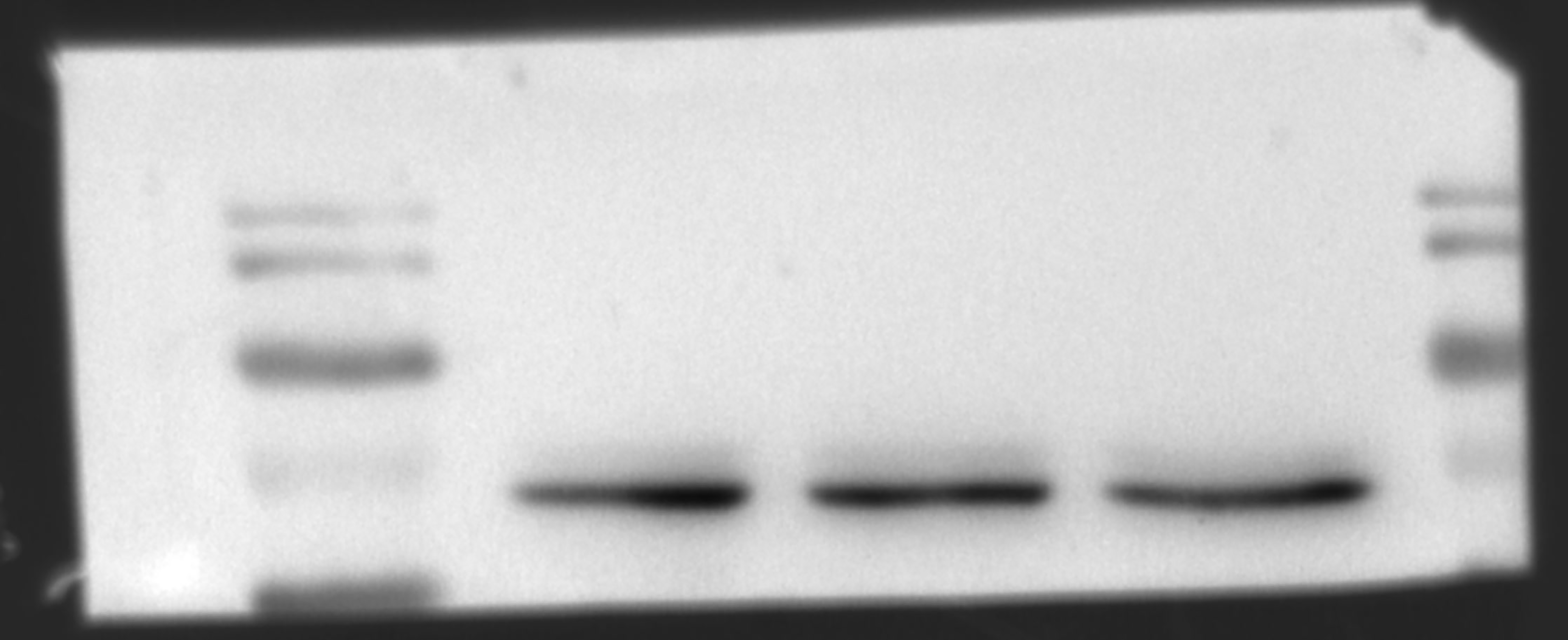

Supplement: Supplementary Figure 3 — Fluorescence minus one (FMO) controls and full stain controls for flow cytometry gating strategy. [file DataSheet3.zip › uncropped and unedited western blot images (Part 2)/p65.tif]

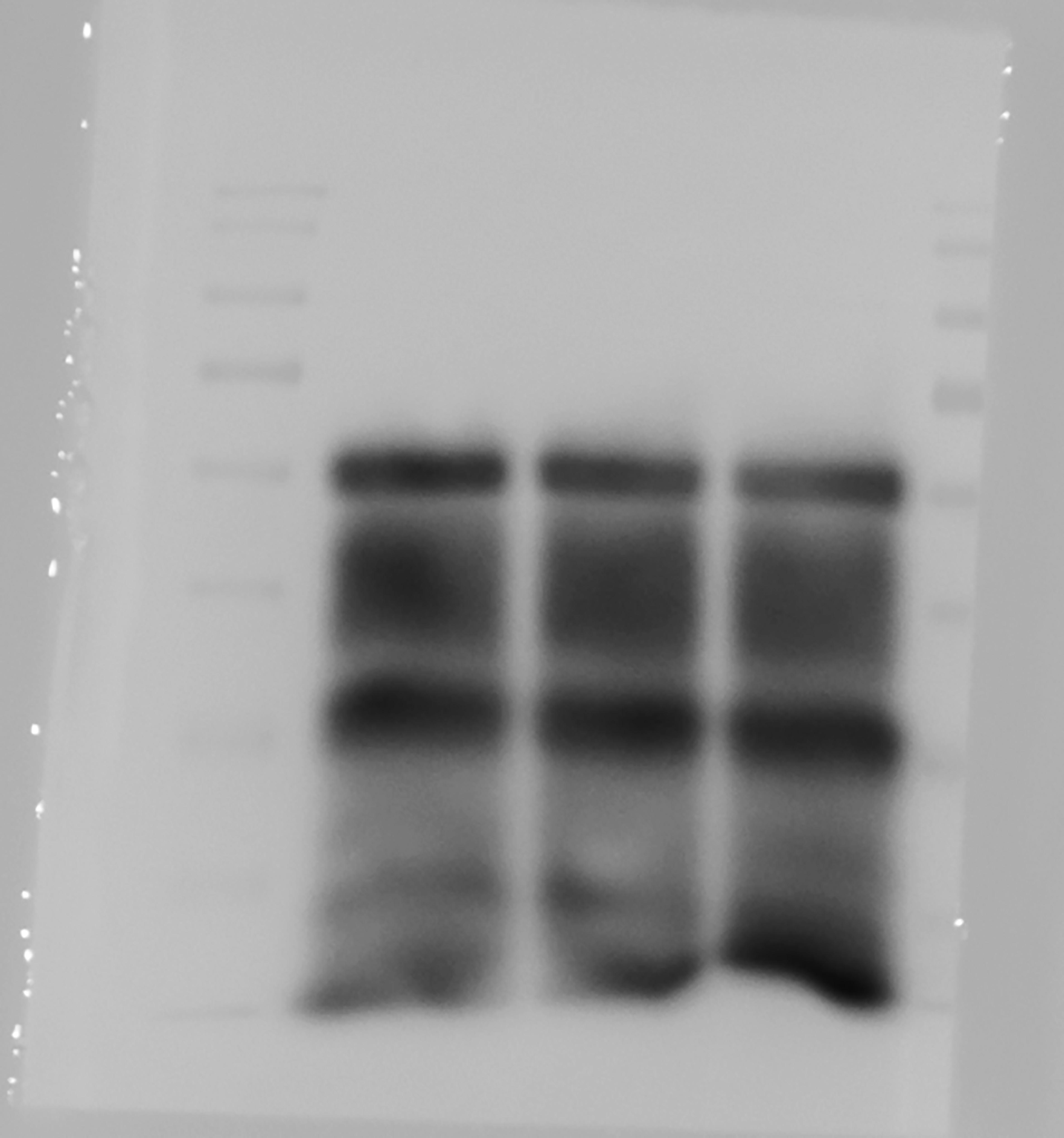

Supplement: Supplementary Figure 3 — Fluorescence minus one (FMO) controls and full stain controls for flow cytometry gating strategy. [file DataSheet3.zip › uncropped and unedited western blot images (Part 2)/IP IB myd88.tif]

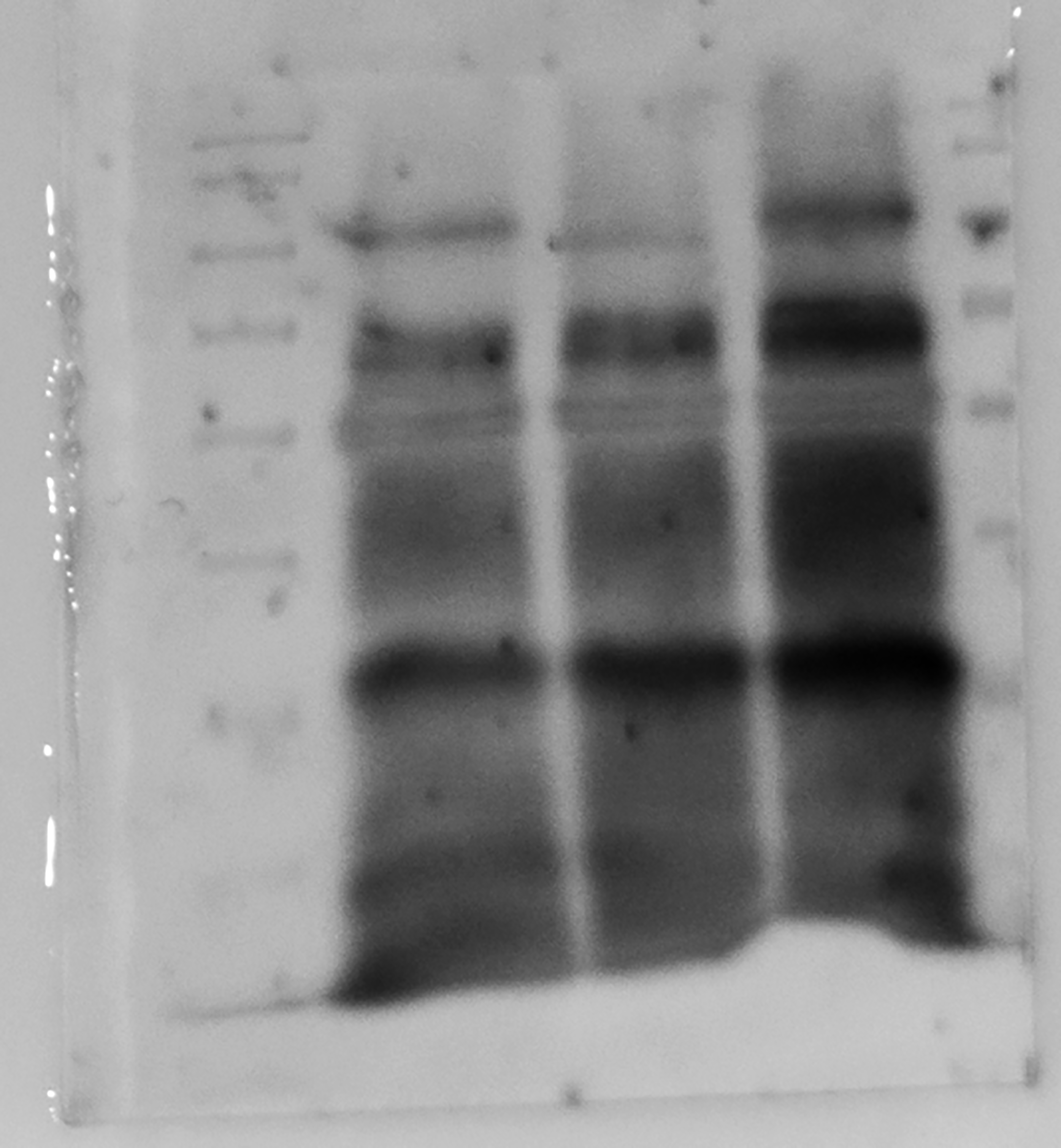

Supplement: Supplementary Figure 3 — Fluorescence minus one (FMO) controls and full stain controls for flow cytometry gating strategy. [file DataSheet3.zip › uncropped and unedited western blot images (Part 2)/myd88 ub.tif]

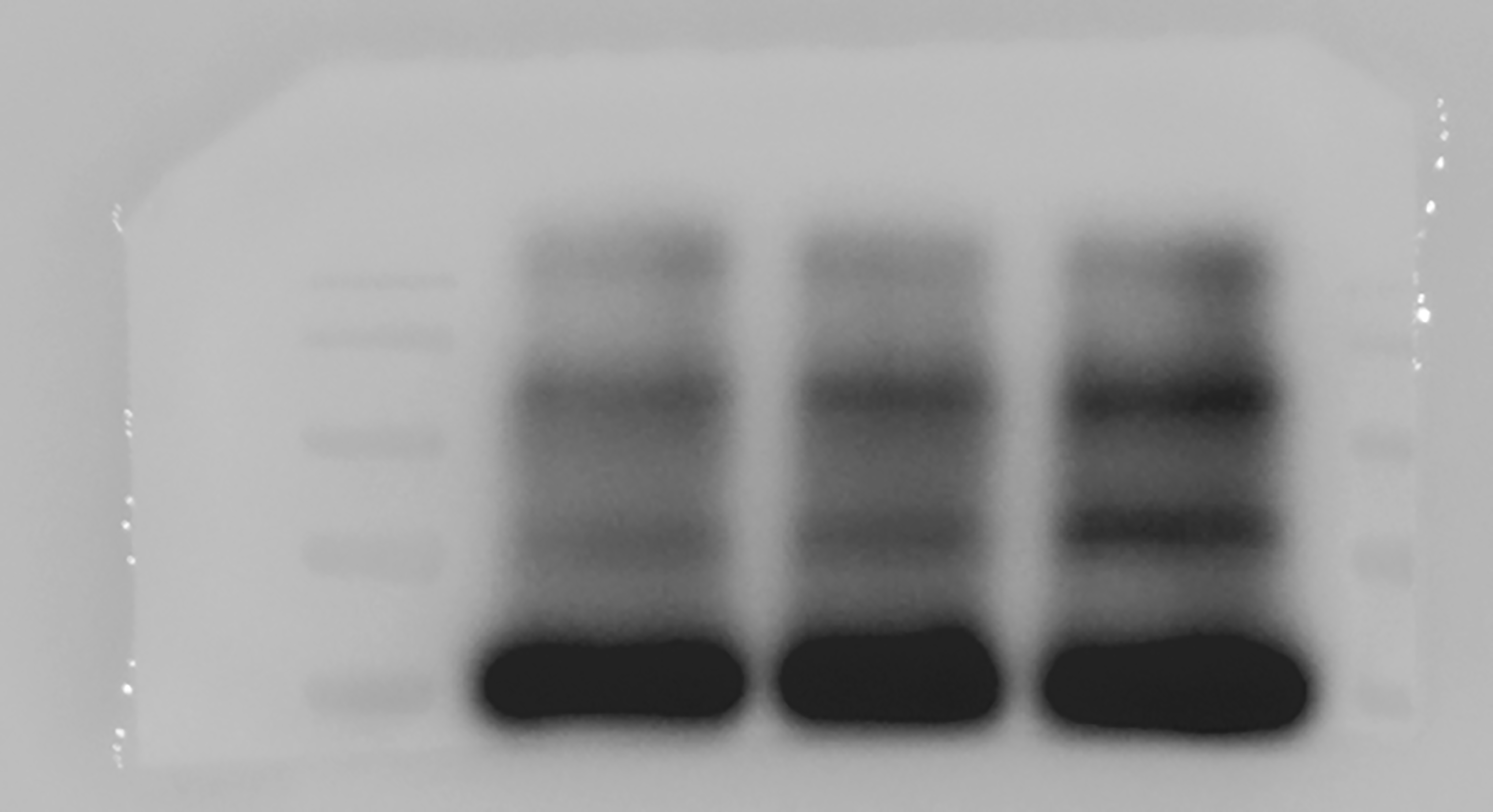

Supplement: Supplementary Figure 3 — Fluorescence minus one (FMO) controls and full stain controls for flow cytometry gating strategy. [file DataSheet3.zip › uncropped and unedited western blot images (Part 2)/IP IB tlr4.tif]

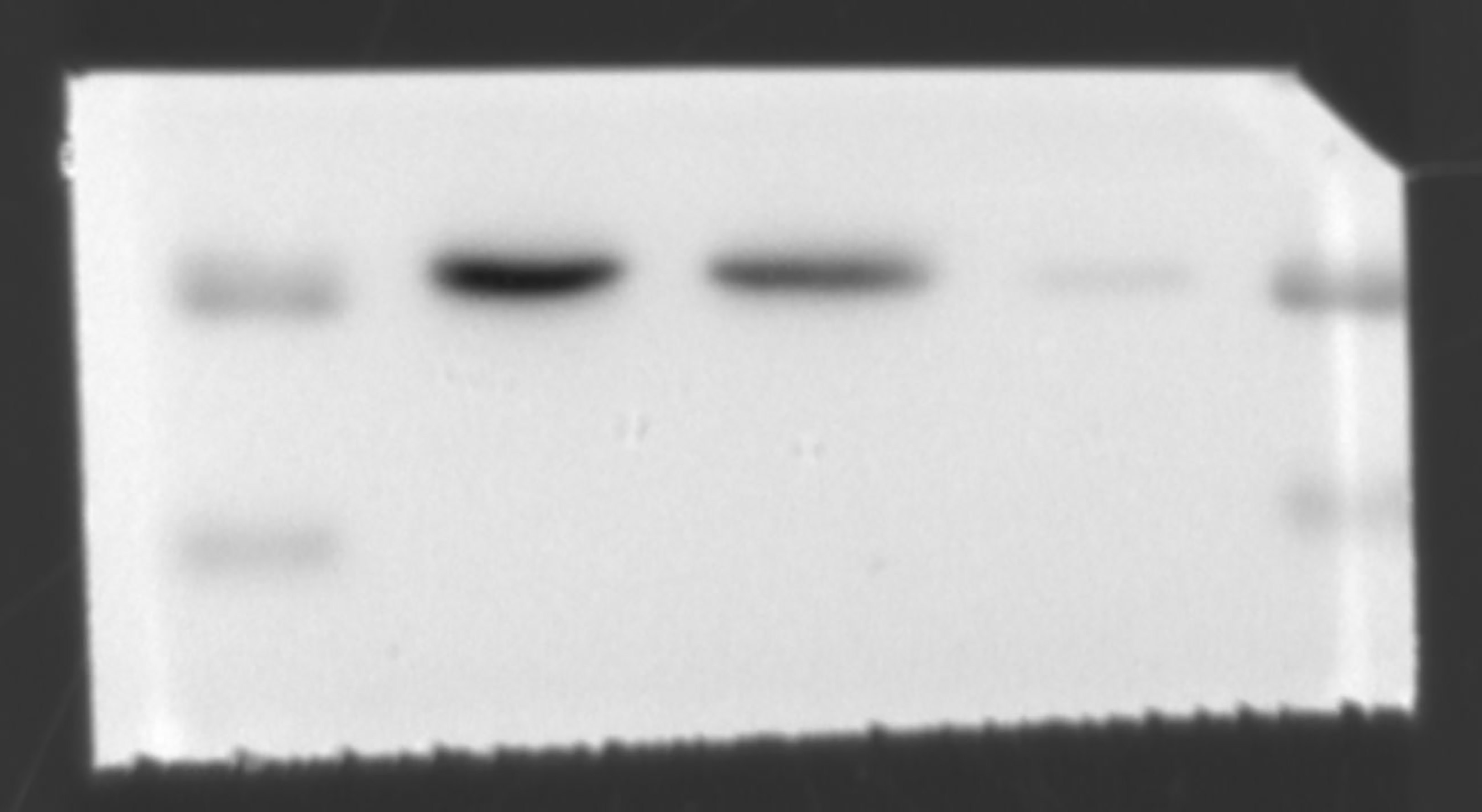

Supplement: Supplementary Figure 3 — Fluorescence minus one (FMO) controls and full stain controls for flow cytometry gating strategy. [file DataSheet3.zip › uncropped and unedited western blot images (Part 2)/myd88 1.tif]

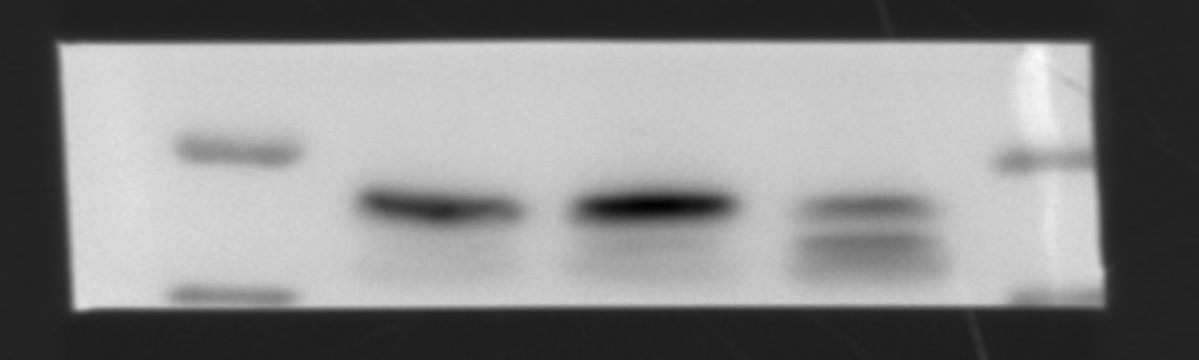

Supplement: Supplementary Figure 3 — Fluorescence minus one (FMO) controls and full stain controls for flow cytometry gating strategy. [file DataSheet3.zip › uncropped and unedited western blot images (Part 2)/ikkb 1.tif]

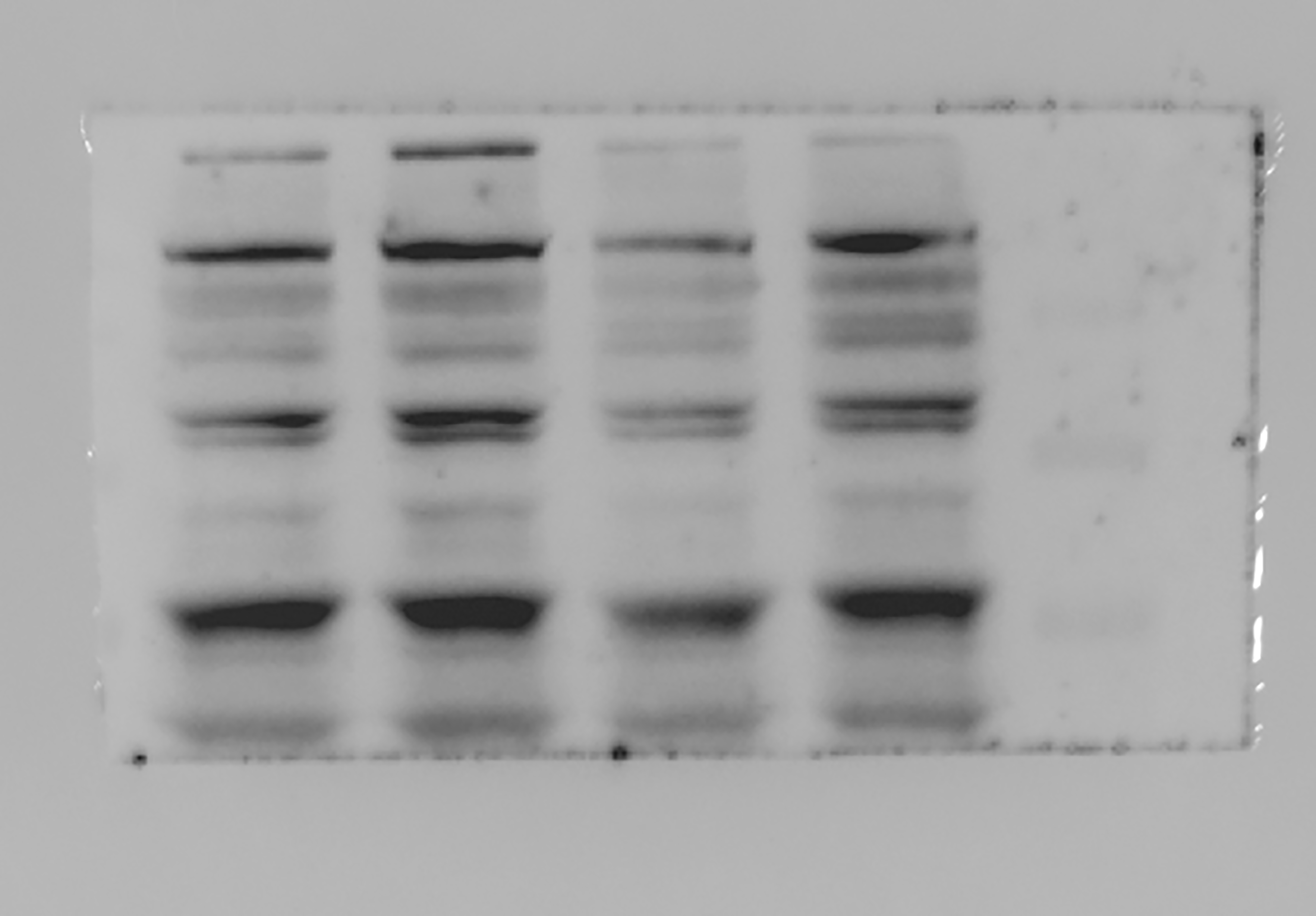

Supplement: Supplementary Figure 3 — Fluorescence minus one (FMO) controls and full stain controls for flow cytometry gating strategy. [file DataSheet3.zip › uncropped and unedited western blot images (Part 2)/tlr4 2.tif]

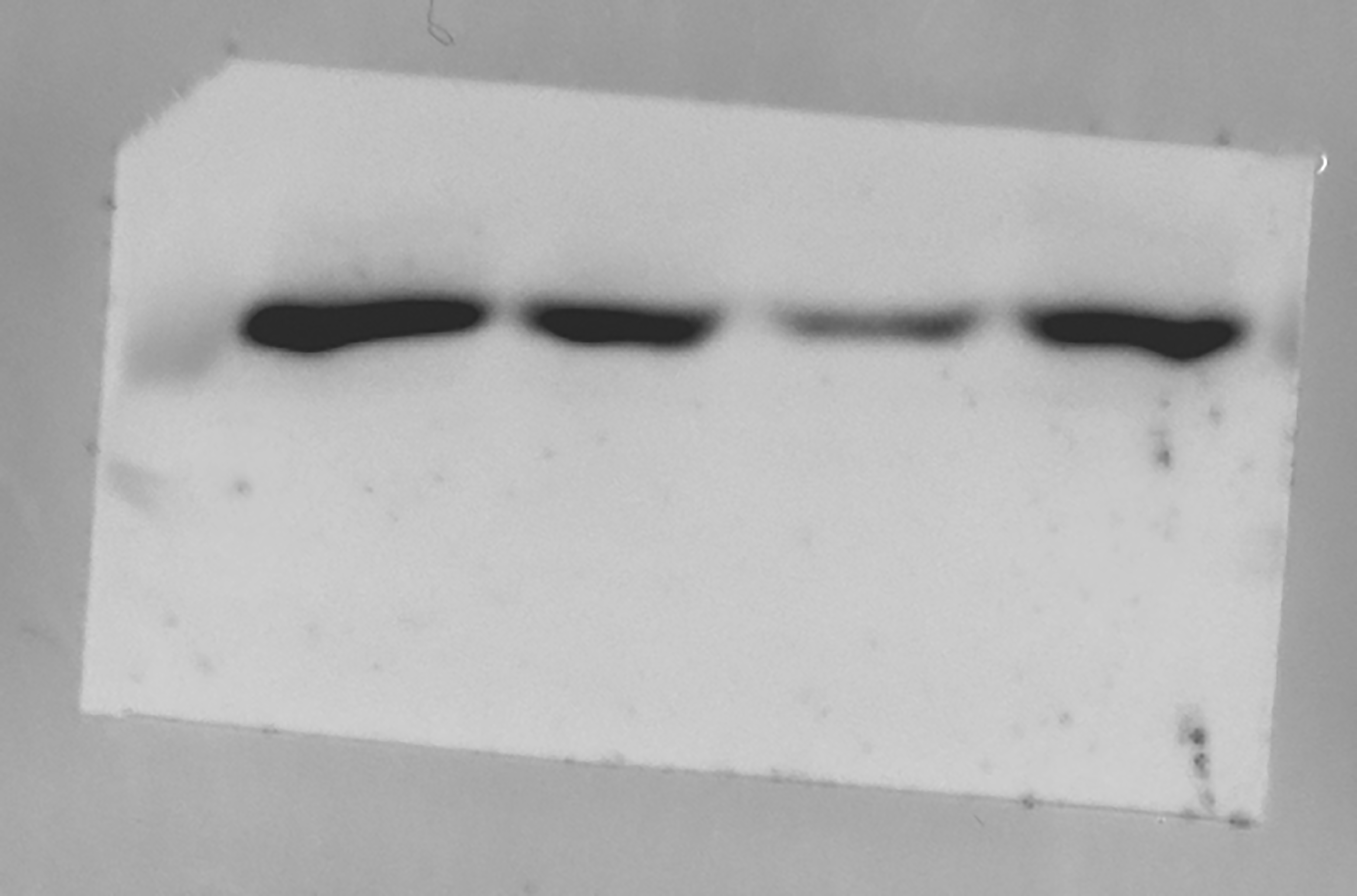

Supplement: Supplementary Figure 3 — Fluorescence minus one (FMO) controls and full stain controls for flow cytometry gating strategy. [file DataSheet3.zip › uncropped and unedited western blot images (Part 2)/myd88 2.tif]

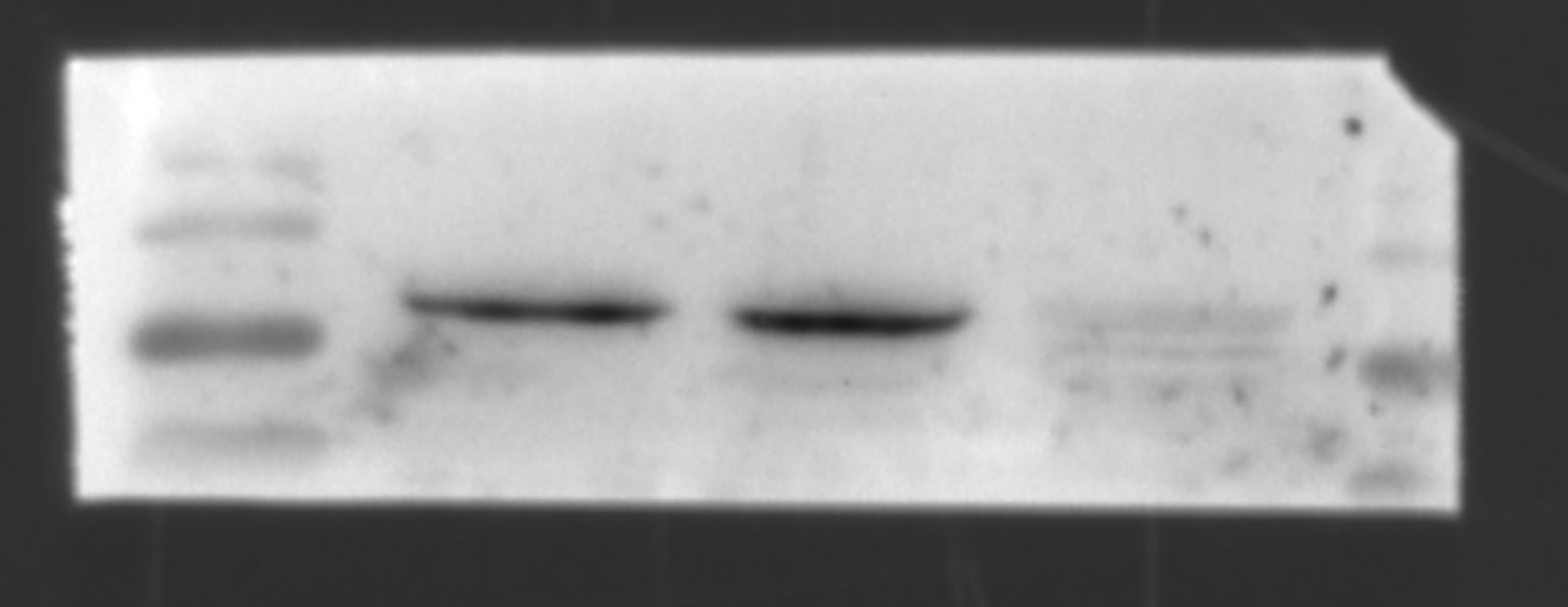

Supplement: Supplementary Figure 3 — Fluorescence minus one (FMO) controls and full stain controls for flow cytometry gating strategy. [file DataSheet3.zip › uncropped and unedited western blot images (Part 2)/tlr4 1.tif]

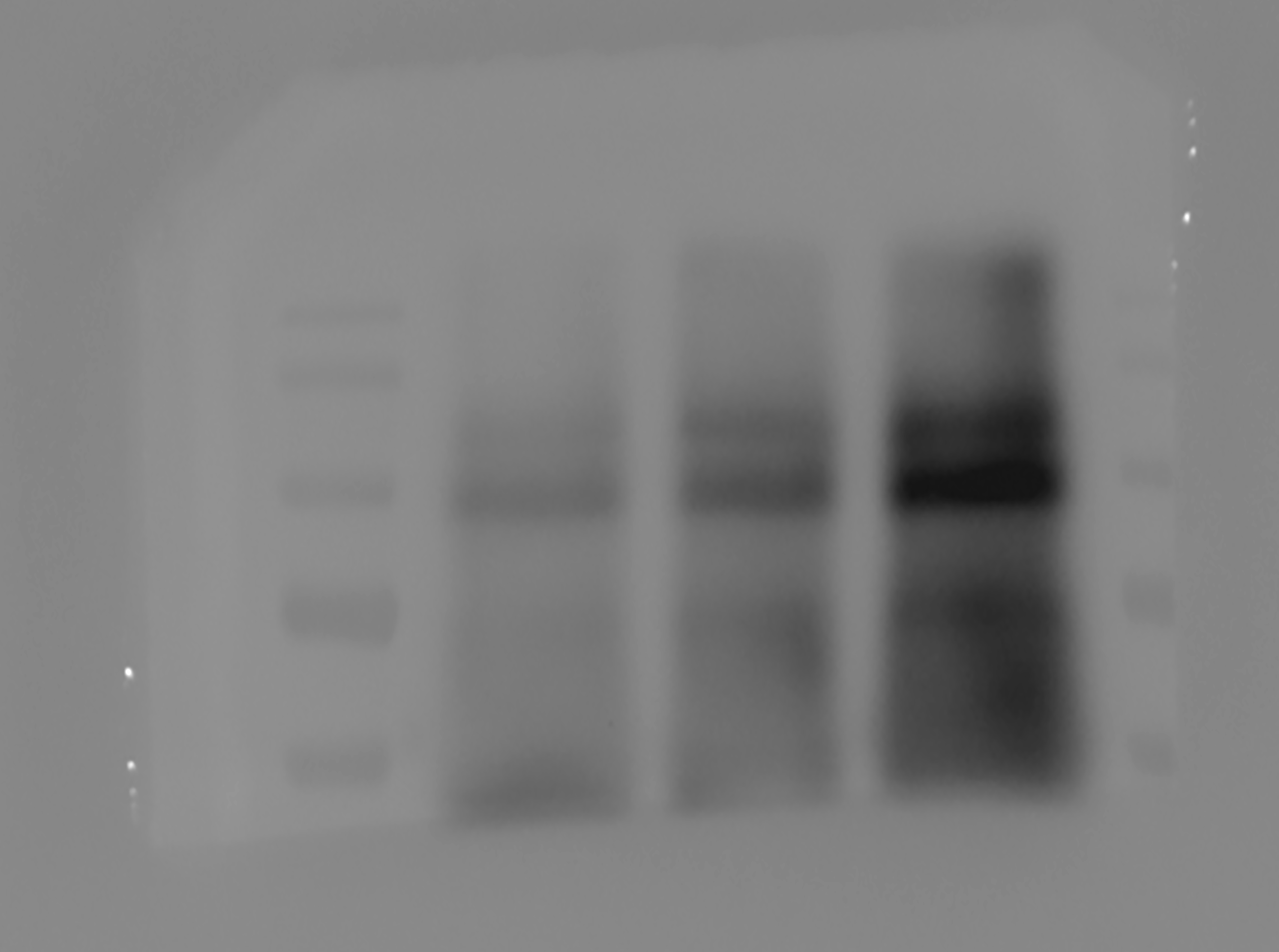

Supplement: Supplementary Figure 3 — Fluorescence minus one (FMO) controls and full stain controls for flow cytometry gating strategy. [file DataSheet3.zip › uncropped and unedited western blot images (Part 2)/tlr4 ub.tif]
