# Supplementary material for: Endothelial HSPA12B regulates myocardial monocyte infiltration and inflammatory activity after myocardial infarction
Source: Front Immunol. 2025 May 15;16:1587898. doi: 10.3389/fimmu.2025.1587898 (PMC12119287; doi:10.3389/fimmu.2025.1587898)
Supplement: Supplementary file 4 [file DataSheet4.docx]

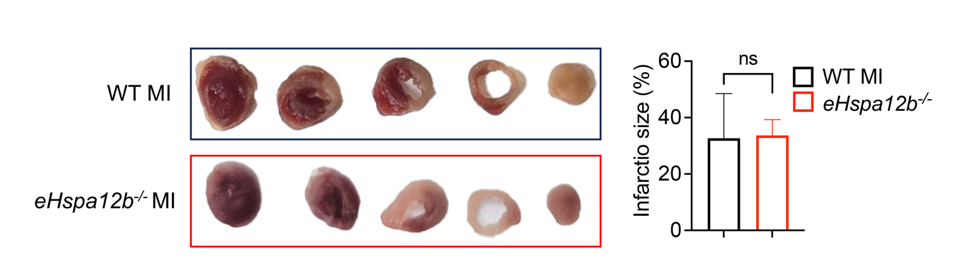


**Supplemental Figure 1.** **Infarct size of wild type and endothelial cell-specific *Hspa12b* knockout myocardial infarction mice.** WT and endothelial cell-specific *Hspa12b* knockout (*Hspa12b^-/-^*) mice were subjected to MI surgery. TTC staining was performed to measure cardiac infarct size. N=3-4. Comparisons of data between groups were made using unpaired t-test.


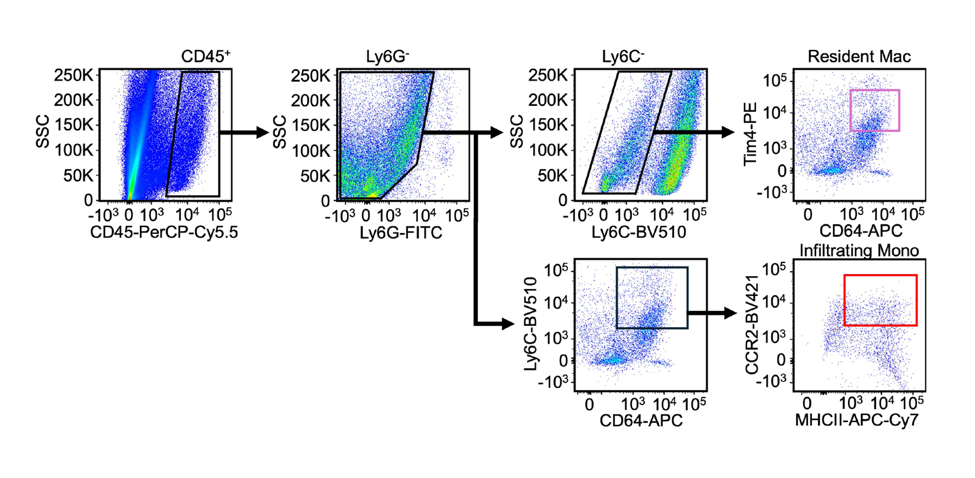


**Supplemental Figure 2. Flow cytometry gating strategies for cardiac macrophages.**

**
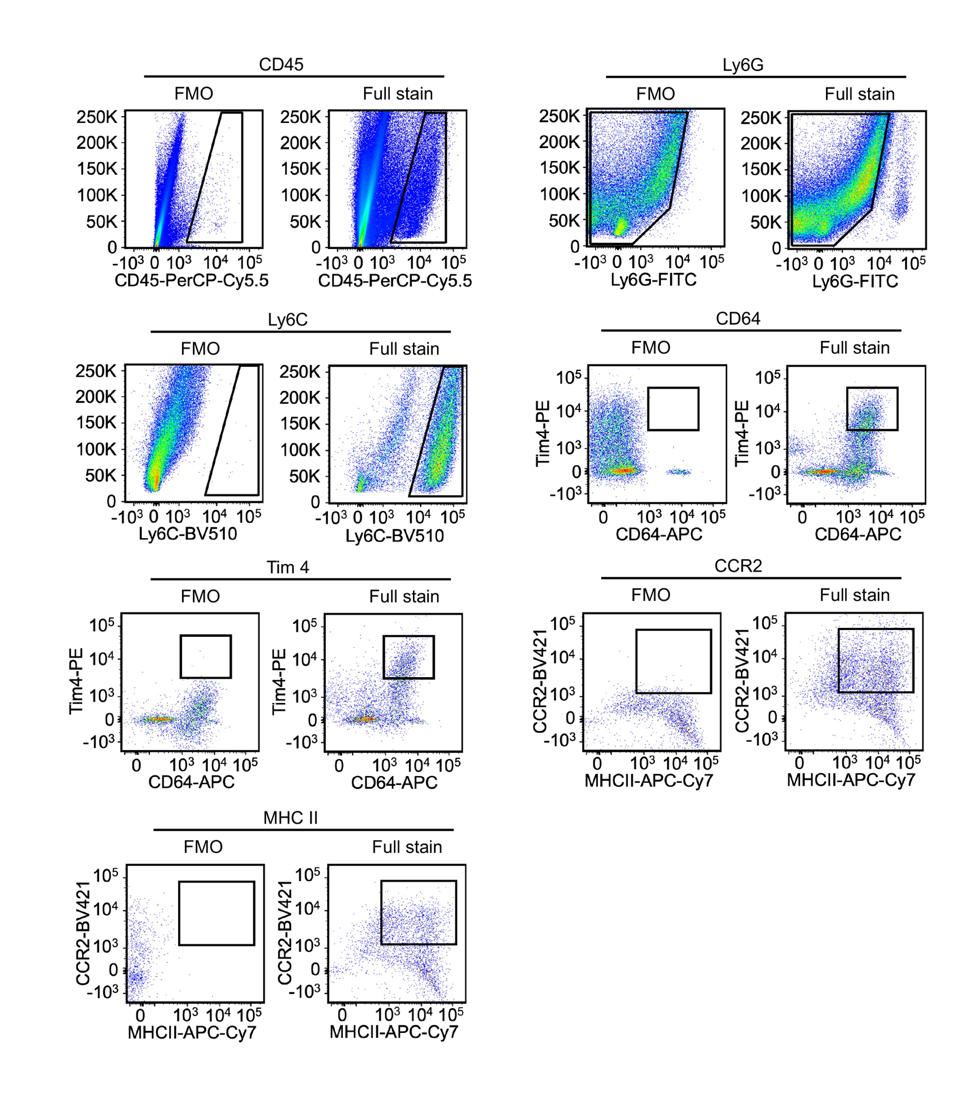
Supplemental Figure 3. Fluorescence minus one (FMO) controls and full stain controls for flow cytometry gating strategy.**
